# Transposon-insertion sequencing reveals NlpD as a vulnerability in intracellular survival and pathogenesis of *Salmonella* 4,[5],12:i:-

**DOI:** 10.1371/journal.ppat.1014640

**Published:** 2026-09-22

**Authors:** Mengping He, Jinju Cai, Huihui Wu, Tianqi Xu, Haijie Zhang, Zhiqiang Wang, Yuan Liu

**Affiliations:** 1 Jiangsu Co-innovation Center for Prevention and Control of Important Animal Infectious Diseases and Zoonoses, College of Veterinary Medicine, Yangzhou University, Yangzhou, China; 2 Jiangsu Key Laboratory of Zoonosis, Yangzhou University, Yangzhou, China; 3 Jiangsu Interdisciplinary Center for Zoonoses and Biosafety, Yangzhou University, Yangzhou, China; University of the Chinese Academy of Sciences, CHINA

## Abstract

*Salmonella* 4,[5],12:i:- has emerged as a leading cause of non-typhoidal salmonellosis globally, yet the mechanisms governing its intracellular survival and pathogenic adaptation remain unclear. Herein, through transposon-insertion sequencing, we identify *nlpD*, a peptidoglycan hydrolase activator essential for maintaining membrane stability and facilitating bacterial division, as a critical gene for *Salmonella* infection. *nlpD*-deficient *Salmonella* exhibits significantly diminished survival in both *in vitro* and *in vivo* settings. Mechanistically, *nlpD* deletion disrupts peptidoglycan and lipopolysaccharide homeostasis, rendering *Salmonella* more vulnerable to oxidative and acidic stresses within phagosomes. Accumulated cell wall components robustly activate the toll-like receptor-mediated NF-κB signaling pathway, driving M1 macrophage polarization and autophagy-lysosomal activation to eliminate intracellular bacteria. An FDA-approved drug screen identifies ketoconazole as an NlpD-targeting compound that reduces bacterial loads in murine models. Our findings establish NlpD as a critical regulator that limits M1-polarized inflammatory responses, highlighting its potential as a therapeutic vulnerability against *Salmonella* 4,[5],12:i:- infection.

## Author summary

*Salmonella* 4,[5],12:i:- is a rapidly spreading global pathogen, but the mechanisms by how it promotes intracellular survival and evades host immune defenses remain elusive. In this study, we identified *nlpD*, a gene required for bacterial cell wall remodeling, as a key factor for *Salmonella* survival within the host. We found that loss of *nlpD* disrupts peptidoglycan and lipopolysaccharide homeostasis, making the bacteria highly sensitive to oxidative and acidic stresses. These cell wall defects activate the host TLR/NF-κB signaling pathway, which shifts macrophages from a permissive M2 state to an activated M1 state. This shift further enhances autophagy-lysosomal degradation to eliminate the intracellular bacteria. We also discovered that ketoconazole can inhibit NlpD and effectively reduce bacterial loads in infected animals. Collectively, our work shows that targeting bacterial cell wall integrity restores host immune clearance and provides a potential therapeutic strategy against *Salmonella* infections.

## Introduction

*Salmonella enterica* serovar Typhimurium (*S.* Typhimurium) is a globally recognized pathogen that causes non-typhoidal salmonellosis, characterized primarily by gastrointestinal symptoms such as diarrhea, fever, and abdominal pain. Non-typhoidal *Salmonella* infections are a major public health concern, causing millions of foodborne illnesses worldwide annually [1]. The monophasic variant 4,[5],12:i:- of *S.* Typhimurium has emerged as a predominant cause of infections in both humans and animals over the past two decades [2,3]. Antimicrobial resistance (AMR) in *Salmonella* substantially exacerbates its public health impact, leading to increased treatment failures and more severe disease outcomes [4]. In 2017, the World Health Organization (WHO) identified antibiotic-resistant *Salmonella* as one of the critical global health threats requiring urgent therapeutic development [5]. Colistin is a critically important antimicrobial for human health, representing one of the last-resort antibiotics against multidrug-resistant (MDR) pathogens [6,7]. The emergence and dissemination of mobile colistin resistance gene *mcr* in *Salmonella* 4,[5],12:i: has undermined the effectiveness of colistin in the clinical setting [8]. Recent genomic surveillance study has revealed the global expansion of the monophasic *Salmonella* Typhimurium sequence type 34 (ST34), which has acquired diverse antimicrobial resistance determinants and disseminated extensively through food-animal reservoirs and human populations. Importantly, a recent study of monophasic *Salmonella* 4,[5],12:i:- isolates recovered from hospitalized patients in China revealed a high prevalence of multidrug resistance and an increasing predominance of ST34, including isolates carrying clinically important resistance determinants such as *mcr-1* and *bla*_CTX-M-14_ [9,10]. These findings underscore the growing clinical burden of MDR ST34 and the increasingly limited therapeutic options for severe infections.

The pathogenicity of *Salmonella* involves a complex interplay between bacterial virulence factors and host immune responses. As a facultative intracellular pathogen, *Salmonella* invades intestinal epithelial cells, replicates within non-phagocytic niches, and persists in macrophage phagosomes across distinct organ systems, necessitating robust adaptation strategies to survive dynamic pH fluctuations and reactive oxygen species. Maintenance of bacterial cell envelope integrity is essential for survival under host-associated stresses. In Gram-negative bacteria, peptidoglycan hydrolases and their regulatory proteins, including LytM-associated systems and NlpD-like activators, coordinate cell wall remodeling, division, and envelope homeostasis [11,12]. However, whether these conserved cell wall regulatory pathways contribute to intracellular survival and virulence of monophasic *Salmonella* remains poorly understood. NlpD is an essential outer membrane lipoprotein that orchestrates bacterial cell division by activating peptidoglycan hydrolysis [13]. Previous studies have linked NlpD to cell envelope remodeling and virulence regulation in several bacterial species. However, whether NlpD contributes to infection of monophasic *Salmonella* 4,[5],12:i:- and whether its functions are dependent on RpoS remain unclear [14,15]. In *S.* Typhimurium, another peptidoglycan hydrolase EnvC promotes cell division by activating the amidases AmiA and AmiC, and its mutant exhibits impaired gut colonization and attenuated inflammatory responses [16]. Despite NlpD’s role in cell division being established, its potential involvement in *Salmonella* virulence has been largely overlooked.

Macrophages function as sentinel innate immune cells that constitute the first line of defense against invading pathogens through phagocytosis and cytokine-mediated inflammatory responses [17]. The pathogenic success of *Salmonella* as an intracellular bacterium depends on its capacity to subvert host innate immunity, particularly by manipulating macrophage polarization and evading autophagic clearance. Although macrophages typically deploy bactericidal M1 phenotypes via TLR4/NF-κB signaling to eliminate pathogens, *Salmonella* has evolved sophisticated mechanisms to reprogram these cells toward a permissive M2-like state [18]. For instance, the SPI-2 type III secretion system effector SteE has been shown to drive M2 polarization through STAT3 activation, thereby promoting persistent colonization [19]. Beyond manipulating polarization, *Salmonella* must counteract host autophagy, an evolutionarily conserved process that targets damaged *Salmonella*-containing vacuoles (SCVs) and cytosolic bacteria for lysosomal degradation. It has been reported that SPI-2 T3SS effectors can modulate host autophagy by altering SCV maturation and mTORC1-associated signaling, allowing *S.* Typhimurium to evade autophagic clearance and survive intracellularly [20]. To ensure intracellular survival, *Salmonella* suppresses autophagic flux by targeting key metabolic sensors such as AMPK and SIRT1 [21]. Despite knowledge of these broad strategies, the specific molecular links between bacterial cell wall integrity and the modulation of host metabolic-autophagy pathways remain poorly characterized. Crucially, it is unclear how cell wall-associated proteins in *S.* 4,[5],12:i:- contribute to immune evasion, and whether these proteins represent viable therapeutic targets.

In this study, we demonstrate that *nlpD* is essential for the intracellular survival and infectivity of *S.* Typhimurium variant 4,[5],12:i:-. Deletion of *nlpD* effectively attenuates *Salmonella* infection in both *in vitro* and *in vivo* settings. Mechanistically, *nlpD* deficiency increased surface exposure of peptidoglycan (PGN) and lipopolysaccharide (LPS), which compromises bacterial resistance to oxidative and acidic stress, and activates host toll-like receptor-mediated NF-κB signaling. This dual effect induces M1 macrophage polarization and enhances autophagy-lysosomal degradation via an AMPK-mTOR dependent manner, thereby diminishing intracellular bacterial survival. Furthermore, combining computational drug screening with experimental validation, we identified the FDA-approved antifungal ketoconazole as a potent NlpD-targeting agent that effectively manages *Salmonella* infections. Our findings highlight that NlpD not only orchestrates the maintenance of a *Salmonella*-favorable M2 microenvironment but also serves as a promising target for pharmacological intervention against intracellular infections.

## Results

### Identification of the essential gene mediating intracellular survival of *Salmonella*

To identify genetic determinants critical for intramacrophage survival, we performed transposon-directed insertion site sequencing (TraDIS) in a clinically isolated *mcr*-positive *S.* 4,[5],12:i:- [22](Fig 1A). The combined library comprised >100,000 clones with mutations in 4,655 of 4,782 genes (97.34% gene-level insertion coverage). And the NZMean values (the average read count across non-zero insertion sites) for the input and output libraries were 235.4 and 198.0, respectively, which significantly exceed the required thresholds for TraDIS (NZMean value > 30), indicating relatively high sequencing coverage across the detected transposon insertion sites (S1A and S1B Fig). High sequencing depth (8,963,185 total reads; ~ 1,925.5 reads per disrupted gene) confirmed random, uniform insertion across the *S*. 4,[5],12:i:- genome (S1B Fig). Subsequently, input and output populations were subjected to TraDIS analysis to identify genes essential for *Salmonella* intracellular survival. Approximately 170 candidate genes exhibited very low insertion representation (*k* ≤ 1) in the output pool. Integrating the transposon insertion-site information for insertion genes with significant changes (Table 1 and S1 Table), we identified four high-confidence candidate essential genes (*nlpD*, *torR*, *apt*, *bcp*) (Fig 1B). These genes were significantly enriched in key functional categories, including two-component signal transduction systems, stress response pathways, lipopolysaccharide synthesis, metalloregulatory transcriptional regulator, and type I/III secretion systems (Fig 1C).

**Table 1:** Transposon insertion sites in genes with significant abundance changes.

Genes	Description	Sites	log_2_FC	p-value
*astA*	arginine N-succinyltransferase	1032	7.89	0.01312
*wzz*	LPS O-antigen length regulator Wzz(fepE)	1134	-0.33	0.01391
*prpE*	propionate-CoA ligase	1884	-0.13	0.01414
*rfaC*	lipopolysaccharide heptosyltransferase RfaC	951	2.43	0.01492
*gntT*	gluconate transporter	1314	-0.43	0.01736
*apt*	adenine phosphoribosyltransferase	549	-7.95	0.01771
*nlpD*	murein hydrolase activator NlpD	1080	-2.54	0.01842
*aac(6’)*	cryptic aminoglycoside N-acetyltransferase AAC(6’)-Iy/Iaa	435	-8.43	0.02021
*phoR*	phosphate regulon sensor histidine kinase PhoR	1293	-0.35	0.0239
*torR*	two-component system response regulator TorR	690	-1.06	0.03091
*tapT*	tRNA-uridine aminocarboxypropyltransferase	678	2.26	0.03767
*flhA*	flagellar biosynthesis protein FlhA	2076	-0.15	0.03904
*bcp*	thioredoxin-dependent thiol peroxidase	468	-3.06	0.03946
*sodA*	superoxide dismutase	618	2.85	0.04344

**Fig 1:**
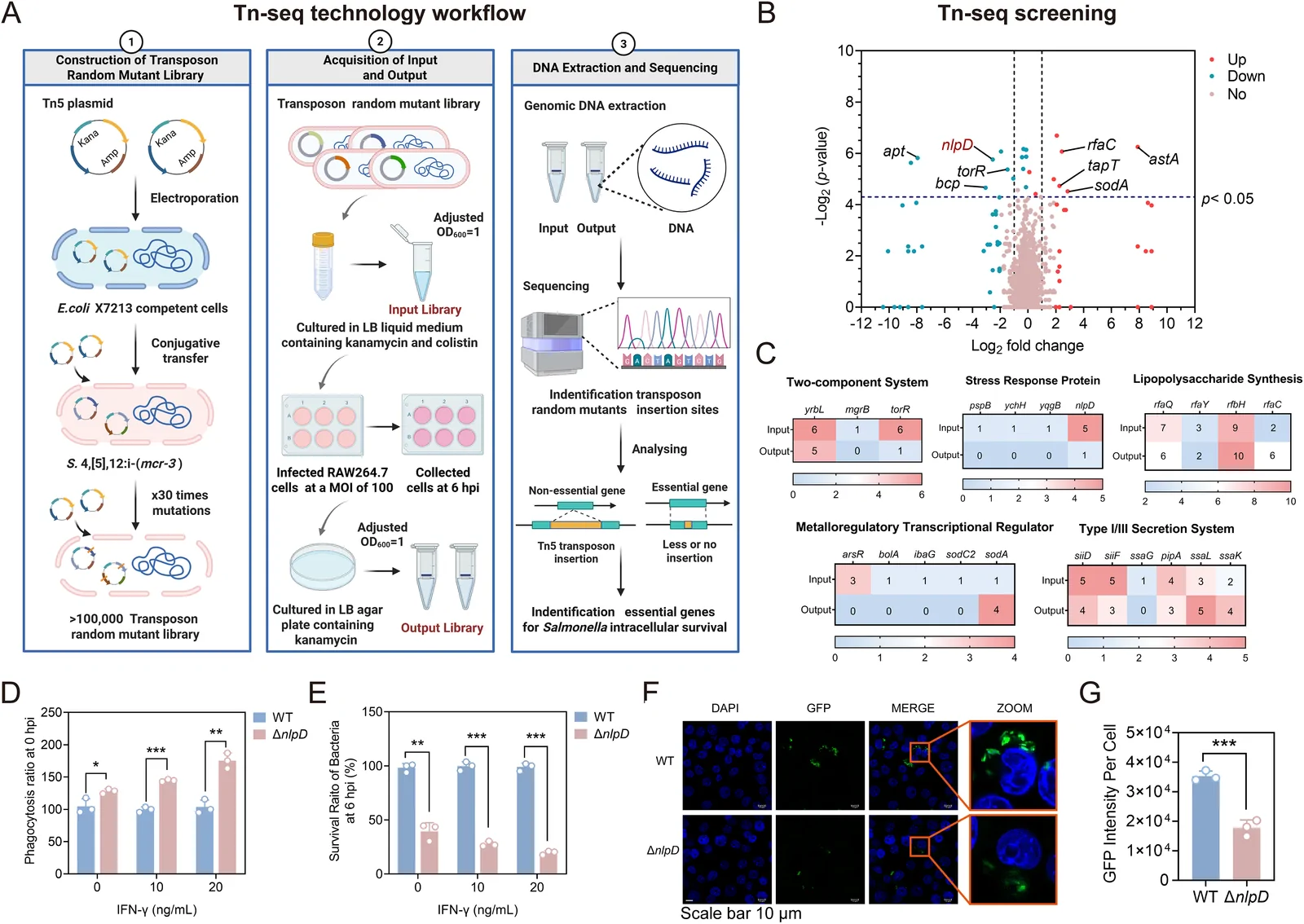
Identification of *nlpD* as an essential virulence gene for intracellular survival of *S*. 4,[5],12:i:- by Tn-seq screening. **(A)** Schematic diagram of the Tn-seq technology workflow for identifying essential genes for the intracellular survival of *Salmonella*. **(B)** Genome-wide distribution of transposon insertion densities by Tn-seq screening, highlighting significant alterations between input and output libraries. Statistical comparisons were performed using Mann-Whitney U test. **(C)** Transposon insertion site densities between input and output libraries within major clustered gene regions. Quantitative analysis of phagocytosis rates **(D)** of RAW264.7 cells infected with WT versus Δ*nlpD* strains and **(E)** intracellular survival capacity of Δ*nlpD* mutant in RAW264.7 cells in comparison with the WT strain after exposure to different concentrations of IFN-γ (0 to 20 ng/mL). Cells were infected with WT and Δ*nlpD* strains at an MOI of 10 for 1 h and incubated for another 6 h. The number of bacteria released from the cells was quantified by plating the lysates. The quantity of Δ*nlpD* was calculated relative to that of the WT (100%). Error bars represent SD; n = 3 independent experiments. Statistical analyses were performed by Two-way ANOVA. *P < 0.05; **P < 0.01; ***P < 0.001. **(F)** Confocal images of intracellular colonization of WT and Δ*nlpD* strains in IFN-γ-activated (20 ng/mL) RAW264.7 cells. Bacterial strains carried GFP-expressing plasmids (green) while host nuclei were counterstained with DAPI (blue). Scale bars: 10 μm. **(G)** Δ*nlpD* showed a significant reduction in relative fluorescence intensity compared to WT-GFP (*P* < 0.001). Error bars represent mean ± SEM; data are representative of three independent experiments. Statistical comparisons were performed using One-way ANOVA.

To validate the essential genes identified by TraDIS, we generated four deletion mutants (Δ*nlpD*, Δ*torR*, Δ*apt* and Δ*bcp*) in *S*. 4,[5],12:i:- (S2A-S2D Fig). Only Δ*nlpD* exhibited significantly reduced intracellular survival (> 60% reduction), whereas Δ*torR*, Δ*apt*, and Δ*bcp* displayed either no effect or minor reductions (< 20%) (S2E and S2F Fig). To confirm the generality of this phenotype, we generated a *nlpD* deletion mutant in *S.* Typhimurium 14028S and *S.* Pullorum WF770, which similarly exhibited enhanced phagocytic uptake and > 50% reduction in intracellular survival relative to wild-type (WT) strains (S3A and S3B Fig; S4A–S4C Fig). These results demonstrated *nlpD* as a critical determinant of *Salmonella* intramacrophage survival. Consequently, we focused subsequent analyses on *nlpD* given its robust and reproducible contribution to pathogenesis.

Given the potent antimicrobial capacity of activated macrophages, we employed IFN-γ at graded concentrations (0, 10, 20 ng/mL) to assess the impact of *nlpD* deletion on *Salmonella* intracellular survival. Quantitative analysis revealed that *nlpD* deletion severely impaired bacterial phagocytosis persistence and intracellular survival capability. Strikingly, in 20 ng/mL IFN-γ-activated macrophages, the *nlpD* mutant showed a 1.75-fold increase in phagocytic uptake and decreased almost 80% survival rate of *Salmonella* compared with the WT strain. These results confirmed that *nlpD* was critical for *Salmonella* intracellular adaptation (Fig 1D and 1E). Consistently, confocal microscopy analysis of IFN-γ-activated macrophages (20 ng/mL) confirmed that *nlpD* deletion significantly reduced *Salmonella* intracellular survival ratio (Fig 1F and 1G), further supporting our findings that *nlpD* is essential for the intracellular survival of *Salmonella*.

### *nlpD* deletion reduces *Salmonella* colonization and host inflammation *in vivo*

Having shown the *nlpD* deletion impaired the *Salmonella* proliferation *in vitro*, we employed an acute *Salmonella* infection murine model to systematically investigated the contribution of *nlpD* for *Salmonella* colonization *in vivo*. Mice were orally inoculated with 10^9^ CFU of either WT or Δ*nlpD* strains. After 24 h, animals were euthanized by cervical dislocation, followed by collection of intestinal and systemic organs for bacterial enumeration and histopathological analysis (Fig 2A). Results demonstrated that *nlpD* deletion exhibited significantly lower colonization levels than the WT strains, with 10- to 100-fold reductions (~1–2 log_10_ CFU/g) in the liver, spleen, colon, cecum, and feces (Fig 2B).

**Fig 2:**
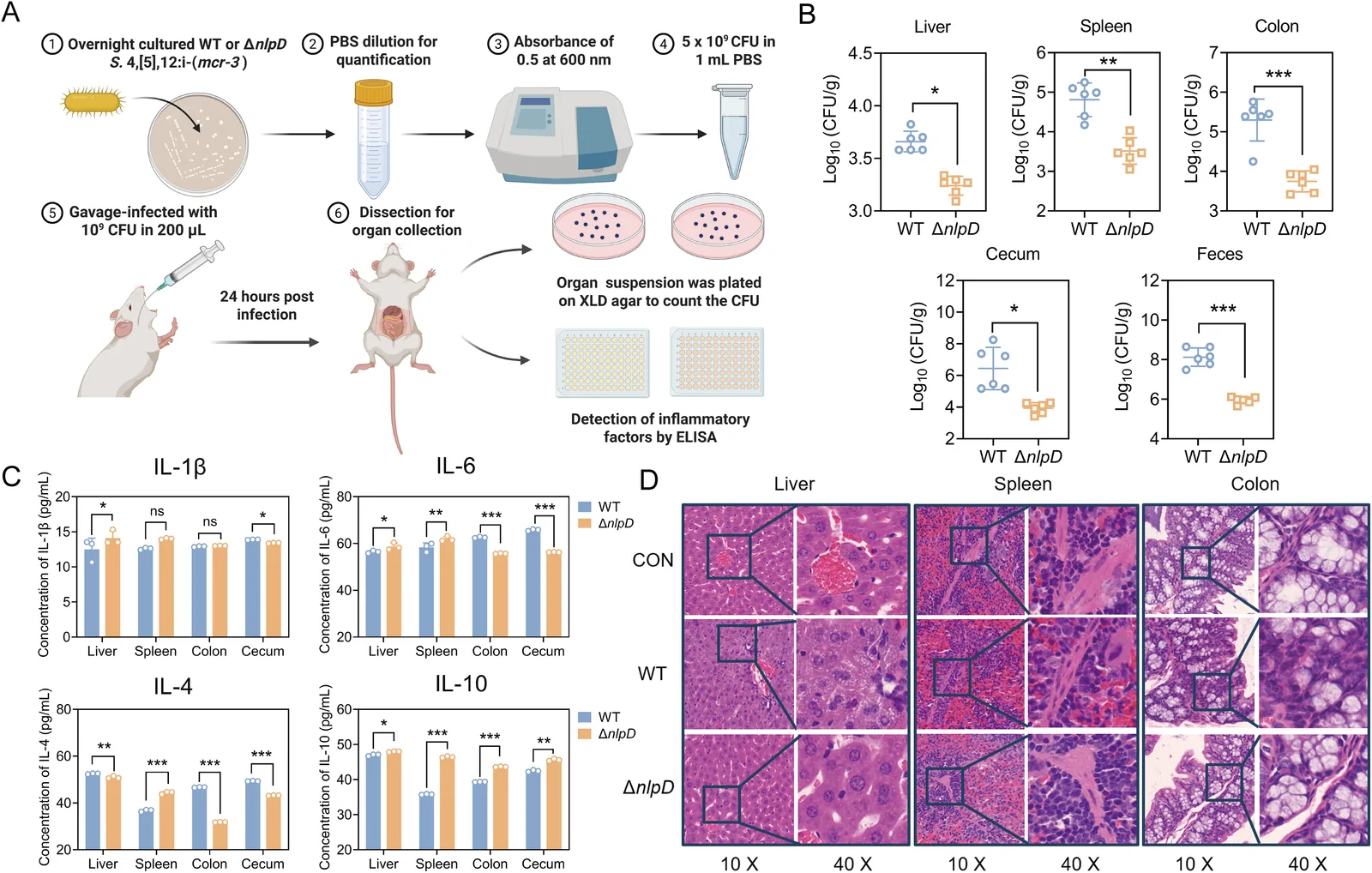
*nlpD* deletion attenuates *in vivo* colonization of *S*. 4,[5],12:i:- and mitigates host inflammation. **(A)** Schematic diagram of a murine oral infection model with *S.* 4,[5],12:i:-. Mice were orally gavaged with either WT strain or Δ*nlpD* mutant (1 × 10^9^ CFU in 200 μL) and analyzed at 24 hpi. **(B)** Comparison of organ colonization properties between the WT and Δ*nlpD* strains. The infection dose was 1 × 10^9^ CFU. Experiments were performed at 24 h post-infection. Each dot represents an organ from an infected mouse. log_10_ (CFU/g) represents the mean of six mice per group. Error bars represent SD. Data were obtained from two independent experiments. Statistical analyses were performed by Mann-Whitney U test. *P < 0.05; **P < 0.01; ***P < 0.001. **(C)** Comparison of inflammatory cytokines expression (IL-1β, IL-6, IL-4, IL-10) in liver, spleen, colon, and cecum of mice at 24 hpi following infection with WT or Δ*nlpD* strains. Each dot represents an organ from an infected mouse. The baseline values were subtracted from all samples. Error bars represent SD; n = 3 independent experiments. Statistical analyses were performed by Two-way ANOVA. *P < 0.05; **P < 0.01; ***P < 0.001; ns: not significant. **(D)** Representative H&E-stained sections of liver, spleen, and colon tissues from mice infected with WT or Δ*nlpD* strains (10 x and 40 x magnification). Compared to the WT-infected group, Δ*nlpD*-infected mice exhibited reduced inflammatory cell infiltration and relatively preserved tissue architecture across all examined organs.

Furthermore, we measured the levels of pro-inflammatory cytokines (IL-1β and IL-6) and anti-inflammatory cytokines (IL-4 and IL-10) in the liver, spleen, colon and cecum using ELISA analysis. In the small intestine (colon and cecum), which serves as the primary infection site, we observed that *nlpD* deletion led to a significant reduction in IL-6 production, accompanied by a marked increase in IL-10 expression. But we did not observe significant differences in IL-1β levels between WT and Δ*nlpD* strains infection mice, which may be attributed to insufficient release of IL-1β at the time of measurement. Moreover, IL-4 exhibited decreased expression in liver, colon, and cecum, while demonstrating increased levels in the spleen. This aligns with findings that goat anti-mouse immunoglobulin D (IgD) antibody (GaMD) stimulation promotes CD4^+^ T cell and basophil expansion in the spleen, leading to rapid IL-4 production [23]. Notably, IL-10 was consistently upregulated across all examined tissues. These findings collectively demonstrated that *nlpD* deficiency effectively attenuates host inflammatory responses during *Salmonella* infection (Fig 2C). Correspondingly, the histopathological analysis revealed profound differences in tissue damage patterns between WT and Δ*nlpD* infections. In mice infected with WT, the liver exhibited karyolysis and vacuolar degeneration, while the spleen displayed neutrophil infiltration. In addition, the colon displayed distinct glandular atrophy and dense inflammatory cell infiltration throughout the tissue architecture. By contrast, Δ*nlpD* strains-infected mice showed completely preserved tissue architecture across all organs, with no pathological changes detected. These results demonstrate that *nlpD* is a virulence-related gene of *Salmonella* and its deletion effectively prevents *Salmonella*-induced multiorgan tissue damage (Fig 2D).

To further verify the critical role of *nlpD* in the pathogenic process of *Salmonella*, we also constructed a *nlpD* knockout mutant in *S.* Typhimurium reference strain 14028S, along with its complemented strain p*nlpD*. Consistent with the findings in *S.* 4,[5],12:i:-, *nlpD* deletion significantly impaired *Salmonella* colonization capability both *in vitro* and *in vivo*. Furthermore, Δ*nlpD* strains markedly alleviated inflammation compared to WT strains of 14028S. Moreover, genetic complementation with p*nlpD* strains fully restored WT strains phenotypes in the Δ*nlpD* mutants (S3 Fig). These results confirm that *nlpD* participates in *Salmonella* pathogenesis and represents an important virulence factor for infection.

### *nlpD* deficiency impairs the antioxidant capacity of *Salmonella*

To elucidate the molecular mechanism underlying *nlpD*-mediated *Salmonella* pathogenesis, we performed transcriptomic analysis of WT strains and Δ*nlpD* mutants of *S.* Typhimurium 14028S during mid-logarithmic growth phase. The principal-component analysis (PCA) demonstrated significant separation between the two experimental groups (Fig 3A). Volcano plot analysis indicated 92 upregulated and 336 downregulated genes in the Δ*nlpD* strains compared to WT strains (Fig 3B). GO enrichment analysis revealed that the top 20 significantly altered pathways were primarily associated with oxidative stress response, cellular response to DNA damage stimulus, response to hydroperoxide, and peptidoglycan-protein cross-linking (Fig 3C). These results indicated that *nlpD* deletion may impair *Salmonella*’s oxidative stress resistance and disrupt peptidoglycan homeostasis, which may contribute to the infectious capabilities of *Salmonella*. Furthermore, we conducted RNA sequencing analysis of WT strains and Δ*nlpD* mutants of 14028S following infected RAW264.7 cells for 6 h. The PCA revealed distinct clustering patterns between the two experimental groups (S5A Fig). Volcano plot analysis identified 1 upregulated and 36 downregulated differentially expressed genes (DEGs) in Δ*nlpD* strains compared to WT strains (S5B Fig). These downregulated genes were predominantly enriched in peroxiredoxin activity, response to hydroperoxide and peroxidase activity (S5C Fig). Collectively, these transcriptomic findings demonstrate that *nlpD* plays a critical role in *Salmonella*’s oxidative stress resistance, likely contributing to its successful host colonization.

**Fig 3:**
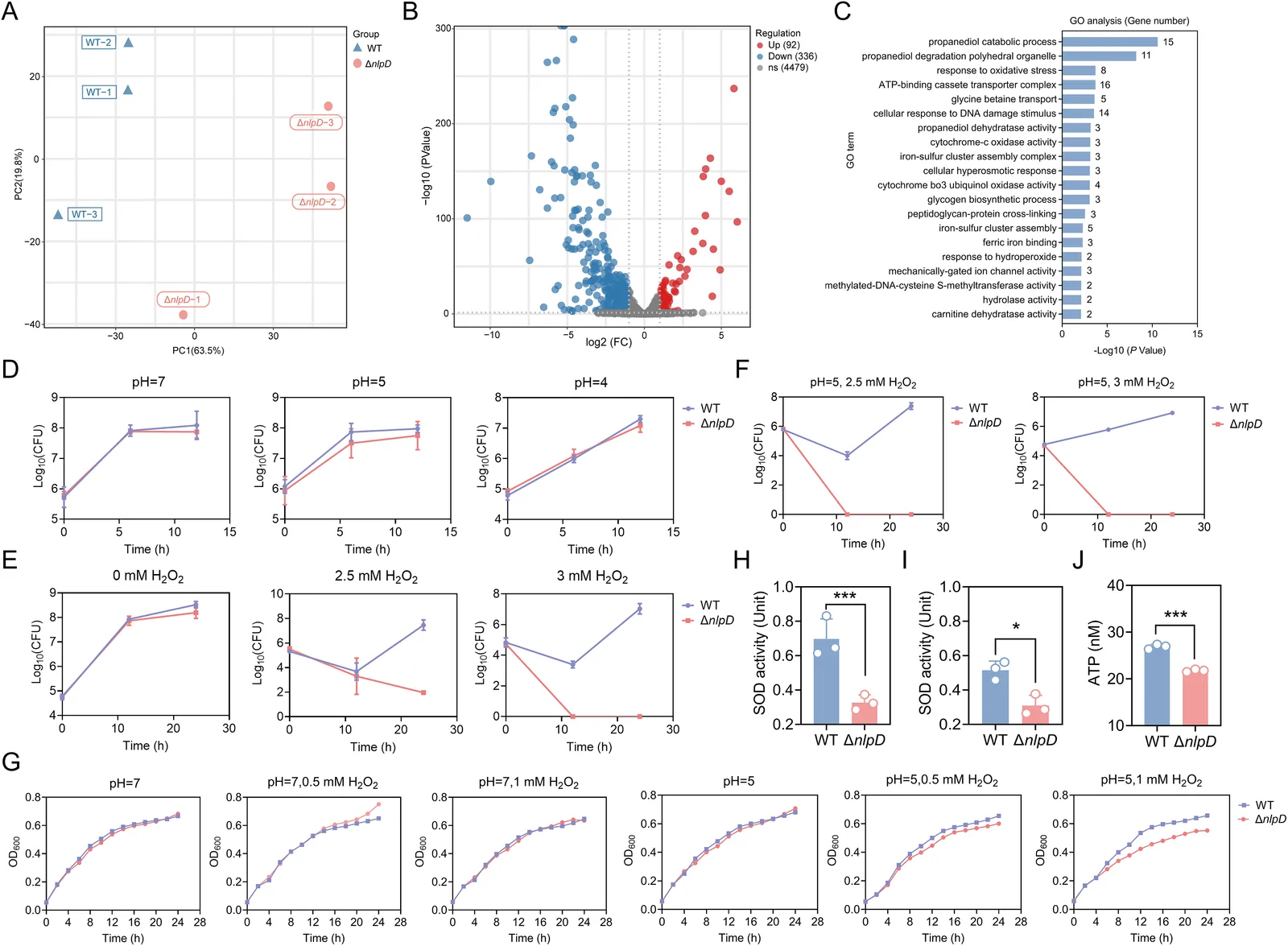
*nlpD* deficiency compromises antioxidant capacity of *S*. 4,[5],12:i:-. **(A)** The principal component analysis (PCA) plot analysis of clustering patterns between WT and Δ*nlpD* strains of 14028S during 6 h cultivation in LB medium using RNA-seq. Each colored dot represents an individual biological sample; n = 3 independent experiments. **(B)** The volcano plot of differentially expressed genes (DEGs) annotated for the WT versus Δ*nlpD* strains of 14028S. Log_2_FC was calculated to account for the direction of expression difference. Genes with log_10_FDR values ≥ 1 and log_2_FC ≥ 1 or log_2_FC ≤ -1 were considered significantly different (red: up-regulated; blue: down-regulated; grey: non-significant). **(C)** GO enrichment analysis revealed that the top 20 significantly altered pathways were primarily associated with oxidative stress response, cellular response to DNA damage stimulus, response to hydroperoxide, and peptidoglycan-protein cross-linking between WT versus Δ*nlpD* strains with a significant threshold of *P* < 0.05. **(D)** Bacterial survival kinetics of WT and Δ*nlpD* strains under varying pH conditions (4, 5, 7) at 6 h and 12 h. Error bars represent SD; n = 3 independent experiments. **(E)** Bacterial survival kinetics by exposing WT and Δ*nlpD* strains to H_2_O_2_ (0, 2.5, 3 mM) in LB broth for 12 h and 24 h. Error bars represent SD; n = 3 independent experiments. **(F)** Time-dependent killing curve of WT and Δ*nlpD* strains under conditions of pH = 5 with different concentration of H_2_O_2_ (2.5, 3 mM) in LB broth for 12 h and 24 h. Error bars represent SD; n = 3 independent experiments. **(G)** Growth curves of WT and Δ*nlpD* strains under varying pH values (5.0 to 7.0) and H_2_O_2_ concentrations (0.5-1 mM) over 24 h (n = 3). Measurements were performed at 2-h intervals. SOD enzyme activity of Δ*nlpD* strains is significantly reduced compared to WT strains at pH = 7 **(H)** and pH = 5 **(I)**. The Total Superoxide Dismutase Assay Kit with WST-8 was used to measure SOD enzyme activity. Error bars represent SD; n = 3 independent experiments. Statistical analyses were performed by Student’s t-test. *P < 0.05; ***P < 0.001. **(J)** Significantly reduced bacterial ATP levels of Δ*nlpD* strains cultured in LB medium for 6 h compared to WT strains. Error bars represent SD; n = 3 independent experiments. Statistical analyses were performed by Student’s t-test. ***P < 0.001.

Given that the phagosome within activated macrophages maintains an acidic pH of 4–5 and exhibits elevated oxidative stress levels [24], which creates significant challenges for intracellular pathogen survival. We compared the survival kinetics of WT and Δ*nlpD* of 14028S under varying pH conditions (pH 5 and 7) and H_2_O_2_ concentrations (2.5 or 3 mM H_2_O_2_). Time-dependent killing curves analysis revealed that the *nlpD* deletion in 14028S significantly inhibited bacterial replication under both H_2_O_2_ stress and macrophage-like conditions, while the p*nlpD* strain restored bacterial survival almost to WT strain’s level (S6A Fig). These phenotypic characteristics were also confirmed in WT and Δ*nlpD* strains of *S.* 4,[5],12:i:-. Similarly, the time-dependent killing curves analysis revealed that *nlpD* deletion did not alter *Salmonella* growth under varying pH conditions (pH 4, 5, and 7; Fig 3D). However, the Δ*nlpD* strain exhibited markedly reduced survival when exposed to oxidative stress (2.5 or 3 mM H_2_O_2_; Fig 3E). Strikingly, Δ*nlpD* strain exhibited rapid cell death under combined acidic (pH = 5) and oxidative stress (2.5 and 3 mM H_2_O_2_) (Fig 3F). These findings demonstrated that *nlpD* deletion significantly impaired *Salmonella* survival under macrophage-like stress conditions. Growth curve analysis further verified that *nlpD* deletion significantly inhibited *Salmonella* replication under simultaneous acidic and oxidative stress conditions (pH = 5 and 1 mM H_2_O_2_; Fig 3G).

Superoxide dismutase (SOD) is a critical metalloenzyme in cellular antioxidant defense systems that catalyzes the dismutation of superoxide radicals (O_2_•^−^) into molecular oxygen (O_2_) and hydrogen peroxide (H_2_O_2_) [25]. To assess the impact of *nlpD* deletion on *Salmonella*’s antioxidant capacity, we next quantified SOD content in both the WT and Δ*nlpD* strains under natural (pH = 7) and acidic (pH = 5) conditions using ELISA. Quantitative analysis revealed that compared to the WT strain, the *nlpD*-deficient strain exhibited 2.3-fold and 1.6-fold reductions in SOD levels at pH = 7 and pH = 5, respectively (Fig 3H and 3I). Consistently, *nlpD* deletion in 14028S strain exhibited lower SOD levels compared to WT strain under both pH = 7 and pH = 5 conditions (S6B Fig), confirming that *nlpD* plays a critical role in maintaining *Salmonella*’s antioxidant defense capacity. Transcriptome analysis also revealed that the *nlpD* deletion disrupted ATP-binding cassette (ABC) transporter complex, which potentially impairing cellular ATP production. The quantification analysis confirmed that *nlpD* deletion significantly reduced bacterial ATP levels (Fig 3J). These findings suggest that *nlpD* deletion compromises bacterial antioxidant capacity, thereby enhancing the sensitivity of *Salmonella* to environmental stressors.

### *nlpD* deletion disrupts cell-envelope homeostasis

The impaired antioxidant capacity and altered ATP levels of the Δ*nlpD* mutants suggested a broader disruption of bacterial physiological homeostasis. Given the role of NlpD in regulating bacterial cell division and the incomplete septation observed in the mutant, we therefore examined whether these defects were accompanied by abnormal accumulation of surface-associated components and altered cell-envelope homeostasis [13]. To characterize the morphological consequences of *nlpD* deletion, we performed scanning electron microscopy (SEM) analysis on mid-log phase cultures (6 h growth in LB medium) of both WT strains and Δ*nlpD* mutants. The results revealed that the *nlpD-*deficient mutants exhibited increased cellular aggregation, with more cells displaying incomplete septation, a hallmark of division impairment. Surface topography analysis demonstrated increased roughness in Δ*nlpD* strains accompanied by abundant extracellular polysaccharide and protein aggregates compared to WT strains (Fig 4A). This suggested that the *nlpD* deletion may lead to abnormal accumulation of surface materials, rendering *Salmonella* more susceptible to environmental stresses. As *nlpD* encodes an outer membrane protein that activates peptidoglycan hydrolases to regulate cell division, we hypothesized that it might play a crucial role in maintaining membrane homeostasis. To test this hypothesis, we examined the membrane fluidity and permeability in both strains. Results confirmed that Δ*nlpD* exhibited significantly decreased membrane fluidity and increased membrane permeability compared to WT strains (Fig 4B-4D), consistent with SEM morphological observations.

**Fig 4:**
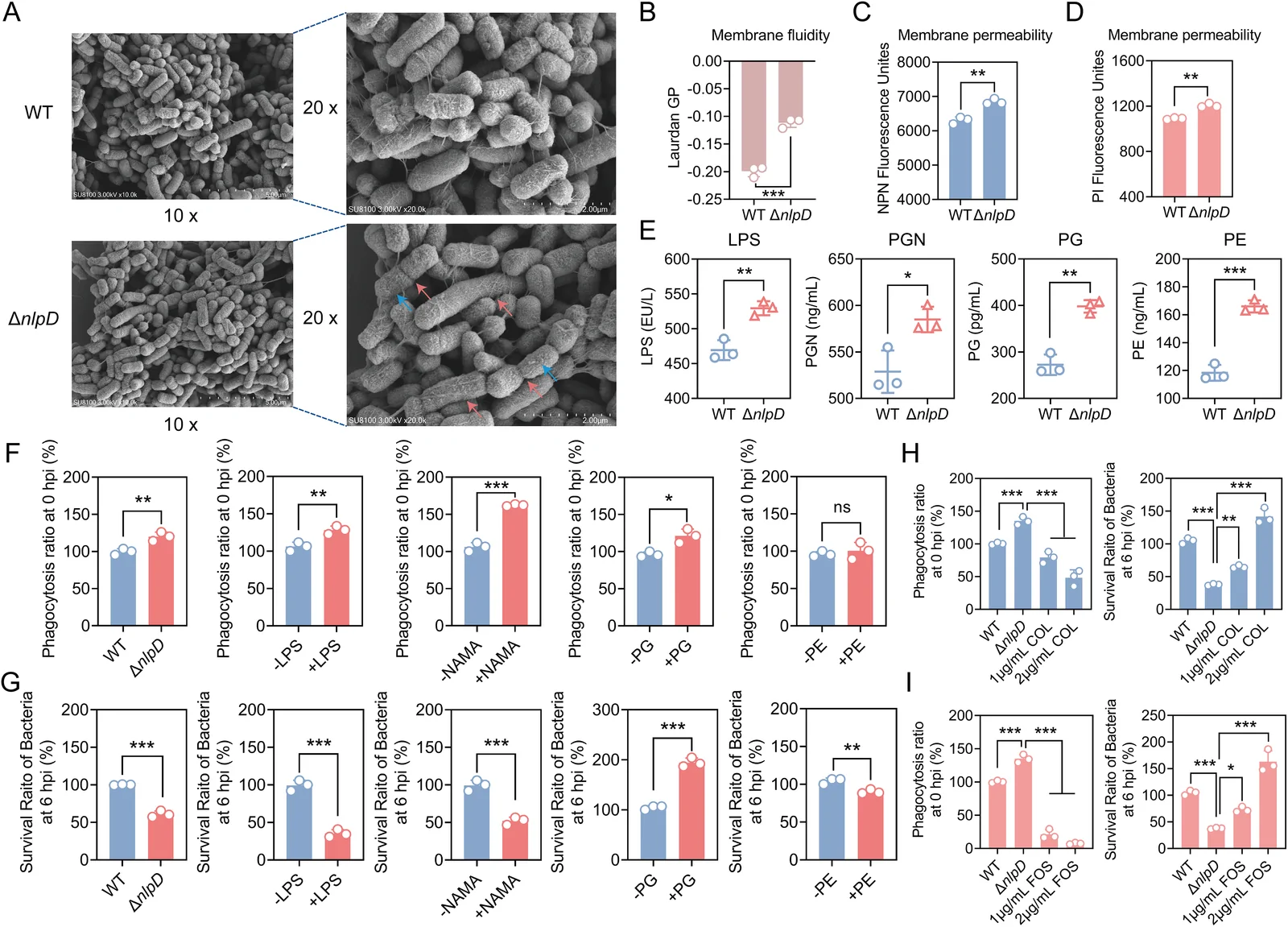
NlpD maintains envelope integrity in *Salmonella.* **(A)** SEM micrographs of WT and Δ*nlpD* strains cultured in LB for 6 h. Surface topography analysis demonstrated increased roughness in Δ*nlpD* strains with more cells displaying incomplete septation. Blue arrows, polysaccharides/proteins aggregate on membranes; red arrows, dividing cells. Scale bars, 5 μM (left) or 2 μM (right). **(B)** Membrane fluidity analysis of WT and Δ*nlpD* mutants using Laurdan GP (10 μM). Δ*nlpD* exhibited significantly decreased membrane fluidity compared to WT strains. Error bars represent SD; n = 3 independent experiments. Statistical analyses were performed by Student’s t-test. ***P < 0.001. Outer membrane permeability between WT and Δ*nlpD* strains, measured by NPN uptake (10 μM) **(C)** and PI uptake (5 μM) **(D)**. Δ*nlpD* exhibited significantly increased membrane permeability compared to WT strains. Error bars represent SD; n = 3 independent experiments. Statistical analyses were performed by Student’s t-test. **P < 0.01. **(E)** Lipopolysaccharide (LPS), peptidoglycan (PGN), phosphatidylglycerol (PG), and phosphatidylethanolamine (PE) levels of WT and Δ*nlpD* mutants cultured in LB medium for 6 h. Δ*nlpD* displayed significantly increased surface exposure of all measured envelope components, including LPS, PGN, PG, and PE, compared with the WT controls. Error bars represent SD; n = 3 independent experiments. Statistical analyses were performed by Student’s t-test. *P < 0.05; **P < 0.01; ***P < 0.001. Quantitative analysis of phagocytosis rates **(F)** of RAW264.7 cells infected with WT versus Δ*nlpD* strains and **(G)** intracellular survival capacity of Δ*nlpD* mutant in RAW264.7 cells in comparison with the WT strain with or without the treatment of LPS (1 μg/mL), NAMA (100 μg/mL), PG (20 μM), and PE (20 μM) for 6 h. The number of bacteria released from the cells was quantified by plating the lysates. The quantity of bacteria was calculated relative to that of the WT or treated without LPS, NAMA, PG and PE (100%). Error bars represent SD; n = 3 independent experiments. Statistical analyses were performed by Student’s t-test. *P < 0.05; **P < 0.01; ***P < 0.001; ns: not significant. Quantitative analysis of phagocytosis rates of RAW264.7 cells (left) and intracellular survival capability of WT and Δ*nlpD* strains (right) treated by COL (1 or 2 μg/mL, **H**) or FOS (1 or 2 μg/mL, **I**). The quantity of bacteria was calculated relative to that of the WT strains (100%). Error bars represent SD; n = 3 independent experiments. Statistical analyses were performed by Student’s t-test. *P < 0.05; **P < 0.01; ***P < 0.001.

To quantitatively assess the impact of *nlpD* deletion on bacterial envelope composition, we identified key surface components using validated ELISA assays. We specifically quantified cell wall-associated lipopolysaccharide (LPS) and peptidoglycan (PGN) levels, alongside membrane phospholipid composition, including phosphatidylglycerol (PG) and phosphatidylethanolamine (PE) contents. The results revealed that Δ*nlpD* displayed significantly increased surface exposure of all measured envelope components, including LPS, PGN, PG, and PE, compared with the WT controls (Fig 4E). Functional complementation studies demonstrated that exogenous administration of specific envelope components differentially destroyed bacterial virulence properties in infected host cells. Specifically, supplementation with LPS and *N*-acetylmuramic acid (NAMA) significantly attenuated both phagocytosis resistance (20% reduction in LPS; 50% reduction in NAMA) and intracellular survival capacity (70% reduction in LPS; 50% reduction in NAMA) of WT strains, exhibiting phenotypes consistent with those of the *nlpD*-deficient strains. However, phospholipid supplementation (PG/PE) showed no concurrent reductions in both anti-phagocytic capacity and intracellular survival of WT strains (Fig 4F and 4G). By contrast, pharmacological reduction of LPS (colistin) or PGN (fosfomycin) restored phagocytosis resistance and intracellular survival in the Δ*nlpD* mutant (Fig 4H and 4I). We analyzed the expression levels of LPS synthesis-related genes via RT-qPCR analysis. The results demonstrated that *nlpD* deletion led to a broad upregulation of genes involved in LPS biosynthesis (S7A Fig).

Given that surface-exposed LPS and PGN recruit pattern recognition receptors TLR4 and TLR2, respectively [26,27], we examined TLR protein levels in RAW264.7 cells. The results showed that Δ*nlpD*-infected cells exhibited elevated TLR4 and TLR2 expression relative to WT-infected cells (S7B and S7C Fig). To validate these findings in a primary cell model, we quantified TLR2 and TLR4 protein levels in bone marrow-derived macrophages (BMDMs) (S7F and S7G Fig). These data support that *nlpD* deletion disrupts cell-envelope homeostasis (increases surface PGN/LPS exposure), thereby enhancing macrophage recognition, phagocytic uptake, and bacterial clearance.

### *nlpD* deficiency triggers M1 macrophage polarization

Since LPS-TLR4 engagement triggers MyD88- and TRIF-dependent signaling cascades, culminating in coordinated MAPK and NF-κB activation that induces proinflammatory gene expression [28,29], we hypothesized that infection with the Δ*nlpD* strains would upregulate phosphorylation of NF-κB pathway proteins, thereby enhancing macrophage activation and bactericidal activity. Consistent with this hypothesis, we observed significantly elevated levels of p-NF-κB (1.4-fold) and p-IκB (1.3-fold) in Δ*nlpD*-infected cells compared to WT-infected cells through western blot analysis (Fig 5A and 5B). We next measured cytokine production in RAW264.7 cells infected with either WT or Δ*nlpD* strains. In line with our previous results, Δ*nlpD* strain triggered significantly higher expression of pro-inflammatory cytokines (IL-6, TNF-α) and reduced levels of anti-inflammatory cytokines (IL-4, IL-10) compared to WT-infected cells (Fig 5C). Meanwhile, we assessed NF-κB pathway activation in BMDMs (S8A and S8B Fig) and quantified cytokine expression. Consistent with the RAW264.7 results, *nlpD* deletion increased p-NF-κB (~3-fold) and p-IκB (~2-fold) in BMDMs. This was accompanied by elevated TNF-α and reduced IL-4 and IL-10 levels, indicating a shift toward pro-inflammatory response (S8C Fig). Similarly, compared with the WT strain, *nlpD* deletion increased BMDM phagocytosis by 30% (S7D Fig). Intracellular *Salmonella* survival was reduced by approximately 60% in BMDMs infected with the Δ*nlpD* mutants (S7E Fig). These findings were consistent with enhanced intracellular bacterial clearance associated with NF-κB activation and the M1-like macrophage phenotype.

**Fig 5:**
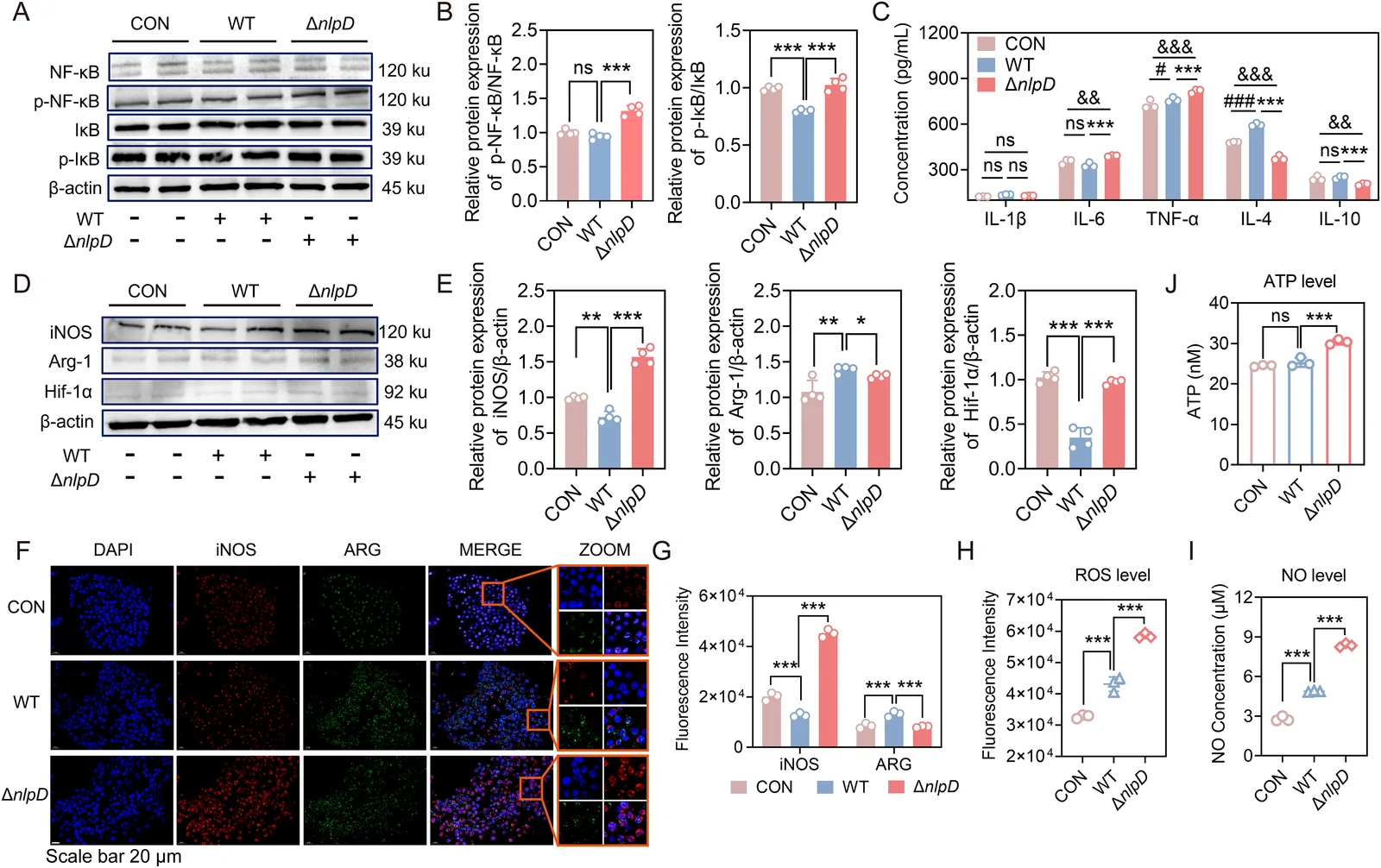
*nlpD* deletion drives M1 polarization of RAW264.7 macrophages. **(A)** Expression levels of NF-κB pathway related proteins (NF-κB, p-NF-κB, IκB, and p-IκB) of uninfected RAW264.7 (CON) and cells infected by WT or Δ*nlpD* mutants for 6 h (n = 4). **(B)** The quantitative analysis of gray value of **(A)** using FIJI software. Data were expressed as percentages normalized by CON group. Error bars represent SD;. n = 4 independent experiments. Statistical analyses were performed by One-way ANOVA. *P < 0.05; ***P < 0.001; ns: not significant. **(C)** Comparison of cytokines expression (IL-1β, IL-6, TNF-α, IL-4, and IL-10) of uninfected RAW264.7 (CON) and cells infected by WT or Δ*nlpD* mutants for 6 h. The baseline values were subtracted from all samples. Error bars represent SD; n = 3 independent experiments. Statistical analyses were performed by Two-way ANOVA. ***P < 0.001; ^#^*P* < 0.05; ^###^*P* < 0.001; ^&&^*P* < 0.01; ^&&&^*P* < 0.001; ns: not significant. **(D)** Protein expression of iNOS (M1 marker), Arg-1 (M2 marker), and Hif-1α of uninfected RAW264.7 (CON) and cells infected by WT or Δ*nlpD* mutants for 6 h (n = 4). **(E)** The quantitative analysis of gray value of **(D)** using FIJI software. Data were expressed as percentages normalized by CON group. Error bars represent SD; n = 4 independent experiments. Statistical analyses were performed by One-way ANOVA. *P < 0.05; **P < 0.01; ***P < 0.001. **(F)** Immunofluorescence detection using an anti-iNOS antibody (red) and anti-Arg-1 antibody (green) of uninfected macrophages (CON) and cells infected by WT or Δ*nlpD* strains at 6 hpi. Nuclei were counterstained with DAPI (blue). Scale bars: 20 μm. **(G)** The quantitative analysis of fluorescent values revealed that Δ*nlpD* significantly increased iNOS fluorescence intensity of cells compared to WT strains. Error bars represent mean ± SEM; data are representative of three independent experiments. Statistical comparisons were performed using Two-way ANOVA. ***P < 0.001. **(H)** ROS, **(I)** NO, and **(J)** ATP level of uninfected macrophages (CON) and cells infected by WT or Δ*nlpD* strains for 6 h. It revealed that infection with Δ*nlpD* strain led to significantly elevated levels of ROS, NO and ATP levels. Error bars represent SD; n = 3 independent experiments. Statistical analyses were performed by One-way ANOVA. ***P < 0.001; ns: not significant.

Accordingly, nuclear translocation of p-NF-κB would induce iNOS expression, leading to increased NO production in macrophages and promoting cells polarization toward the M1 phenotype [30,31]. Since M1-polarized macrophages enhance bacterial clearance, we analyzed the polarization-related proteins expression. Notably, Δ*nlpD* strains upregulated the expression of M1 polarization markers, including iNOS (2-fold) and hypoxia-inducible factor HIF-1α (3.3-fold), compared to cells infected with WT strains (Fig 5D and 5E). Immunofluorescence staining revealed that *nlpD* deletion significantly increased iNOS fluorescence intensity of cells (Fig 5F and 5G), confirming M1 macrophage polarization. M1-polarized macrophages typically exhibit increased production of reactive oxygen species (ROS) and nitric oxide (NO), which contribute to their enhanced bactericidal activity [32]. Consistent with this paradigm, infection with Δ*nlpD* strain led to significantly elevated levels of both ROS and NO (Fig 5H and 5I). These increases correlated with diminished bacterial antioxidant capacity, leading to more effective microbial clearance. Furthermore, intracellular ATP levels were significantly induced in Δ*nlpD*-infected cells than in WT-infected cells (Fig 5J). These findings indicate an enhanced metabolic activity associated with macrophage-mediated antibacterial responses. In BMDMs, Δ*nlpD* elicited an M1 polarization profile relative to WT, characterized by elevated M1 markers (iNOS and HIF-1α) and diminished M2-associated Arg-1 expression (S8D and S8E Fig). These findings corroborate that *nlpD* deletion promotes M1-like macrophage polarization.

### *nlpD* deletion promotes autophagy in *Salmonella*-infected macrophages

While autophagy serves as a critical downstream effector in innate and adaptive immunity by eliminating intracellular pathogens, *Salmonella* has evolved sophisticated mechanisms to evade autophagic clearance and exploit host pathways for intracellular survival [21,33,34]. We therefore postulated that *nlpD* may contribute to *Salmonella* virulence by negatively regulating autophagic clearance pathways. To investigate the potential role of autophagy in Δ*nlpD*-mediated bacterial clearance, we analyzed key autophagy markers, including microtubule-associated protein 1 light chain 3 (LC3) and SQSTM1/p62. Also, autophagic flux can be quantitatively evaluated by analyzing the dynamic interplay between LC3 lipidation and p62/SQSTM1 degradation, and increased LC3-II conversion coupled with reduced p62 levels provides definitive evidence of functional autophagic progression [33,35]. Compared with WT infection, Δ*nlpD* infection increased the LC3-II/LC3-I ratio by approximately 2.3-fold (Fig 6A and 6B). In parallel, p62 levels decreased by approximately 40%. These changes were consistent with enhanced autophagic activity in Δ*nlpD*-infected cells. Additionally, the autophagy initiator Beclin 1 showed significantly higher expression in Δ*nlpD*-infected cells relative to WT-infected cells (Fig 6A and 6C), reinforcing the evidence for augmented autophagic activity. We further validated these findings in BMDMs and found that *nlpD* deletion similarly increased autophagic flux, as indicated by an elevated LC3-II/LC3-I ratio (~1.3-fold) and reduced p62 protein levels (~1.2-fold) (S8F and S8G Fig). Likewise, *nlpD* deletion also upregulated the autophagy initiator Beclin 1 (~1.3-fold) (S8F and S8G Fig), consistent with our observations in RAW264.7 cells.

**Fig 6:**
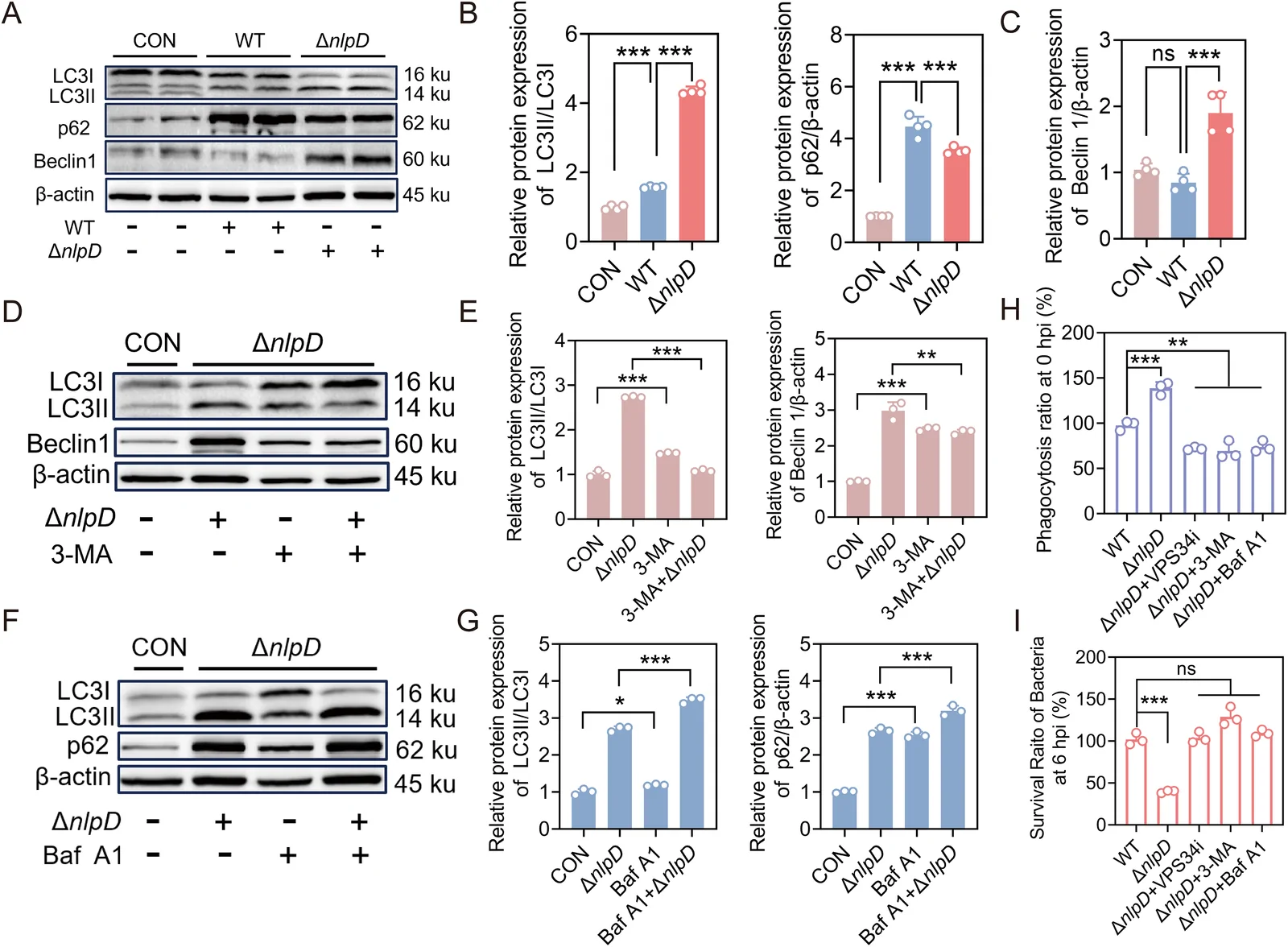
*nlpD* deletion enhances autophagic clearance of *Salmonella* in macrophages. **(A)** Immunoblot analysis of autophagy markers (LC3, p62, and Beclin 1) of uninfected RAW264.7 (CON) and cells infected by WT or Δ*nlpD* mutants of *S.* 4,[5],12:i:- for 6 h (n = 4). **(B and C)** The quantitative analysis of gray value of **(A)** using FIJI software. Data were expressed as percentages normalized by CON group. Error bars represent SD; n = 4 independent experiments. Statistical analyses were performed by One-way ANOVA. ***P < 0.001; ns: not significant. **(D)** LC3 and Beclin 1 protein expression of uninfected RAW264.7 (CON) and cells infected by Δ*nlpD* mutants-infected macrophages pre-treated with or without 3-MA (5 μM) for 6 h (n = 3). **(E)** Quantitative analysis of gray value of **(D)** using FIJI software. Data were expressed as percentages normalized by CON group. Error bars represent SD; n = 3 independent experiments. Statistical analyses were performed by One-way ANOVA. **P < 0.01; ***P < 0.001. **(F)** LC3 and p62 protein expression of uninfected RAW264.7 (CON) and cells infected by Δ*nlpD* treated with or without Baf A1 (200 nM) for 6 h (n = 3). **(G)** Quantitative analysis of gray value of **(F)** using FIJI software. Data were expressed as percentages normalized by CON group. Error bars represent SD; n = 3 independent experiments. Statistical analyses were performed by One-way ANOVA. *P < 0.05; ***P < 0.001. Quantitative analysis of phagocytosis capability **(H)** of RAW264.7 cells and **(I)** intracellular survival rates of WT and Δ*nlpD* strains treated by VPS34i (25 nM), 3-MA (5 μM) or Baf A1 (200 nM). The quantity of bacteria was calculated relative to that of the WT strains (100%). Error bars represent SD; n = 3 independent experiments. Statistical analyses were performed by One-way ANOVA. **P < 0.01; ***P < 0.001; ns: not significant.

To further confirmed the role of *nlpD* deletion in autophagy activation, we utilized two stage-specific inhibitors. Specifically, 3-methyladenine (3-MA) was employed to block autophagosome formation during the initiation phase, while bafilomycin A1 (Baf A1) was used to inhibit autophagosome-lysosome fusion in the termination phase [36]. In Δ*nlpD*-infected cells, 3-MA treatment reduced the LC3-II/LC3-I ratio from 2.8-fold to 1.0-fold compared with the untreated group (Fig 6D and 6E). Beclin 1 expression was also reduced from 3.0-fold to 2.1-fold. These changes indicated effective suppression of autophagosome formation by 3-MA. Baf A1 treatment, which blocks lysosomal acidification and subsequent autophagosome-lysosome fusion, caused marked accumulation of both LC3-II (from 2.5- to 3.3-fold) and the autophagy substrate p62 (from 2.5- to 3.0-fold) compared to untreated group (Fig 6F and 6G). The Baf A1-induced accumulation of autophagic markers (LC3-II and p62) demonstrated that Δ*nlpD* infection enhanced autophagosome formation and subsequent blockade of degradation. Together, these results suggested that *nlpD* deletion promoted autophagy progression through coordinated activation of both initiation and termination phases.

To investigate the role of autophagy in limiting *Salmonella* intracellular survival, we assessed both antiphagocytic capacity and intracellular survival rate of WT and Δ*nlpD* strains after treatment with three stage-specific autophagy inhibitors, including VPS34 inhibitor (VPS34i), 3-MA, and Baf A1. The pharmacological inhibition of autophagy, both at the early stage (using VPS34i and 3-MA) and at the late stage (using Baf A1), significantly reversed the enhanced phagocytic clearance and restored the intracellular survival of the Δ*nlpD* strain to levels comparable to those seen in WT-infected cells (Fig 6H and 6I).

To exclude potential off-target effects of pharmacological autophagy inhibitors on phagocytosis or other cellular processes, we genetically silenced the autophagy initiator Beclin 1 using siRNA (S2 Table). Beclin 1 protein expression was efficiently knocked down after 48 h of siRNA transfection (S9A and S9B Fig). Importantly, Beclin 1 silencing significantly restored both elevated phagocytic uptake and reduced intracellular survival in Δ*nlpD* but not WT strains (S9C Fig). These results provide independent genetic evidence that autophagy is activated by *nlpD* deficiency and drives enhanced intracellular killing, rather than representing an artifact of pharmacological inhibition.

### *nlpD* deficiency enhances lysosomal activity of infected cells

Following pathogen engulfment, autophagosomes undergo fusion with acidic lysosomes to generate degradative autolysosomes for efficient pathogen clearance [37]. To investigate whether *nlpD* deletion induces lysosomal activation in infected cells, we quantified two lysosomal markers: LAMP1 (lysosomal membrane glycoprotein) and ATP6V0D1 (v-ATPase subunit essential for lysosomal acidification) [38,39]. Notably, *nlpD* deletion significantly upregulated the expression of LAMP1 by 1.75-fold and ATP6V0D1 proteins by 2.2-fold relative to WT strains-infected cells (Fig 7A and 7B). Immunofluorescence analysis showed significantly stronger LAMP1 signals in Δ*nlpD*-infected cells than in WT-infected cells (Fig 7C and 7D). This observation was consistent with enhanced lysosomal activation. To investigate the upstream regulation of autophagy activation, we examined the AMPK-mTOR pathway, a master regulator of autophagic flux. Remarkably, Δ*nlpD* infection increased p-AMPK levels while inhibiting p-mTOR activity, as well as decreased p70S6K1 (mTOR downstream protein) (Fig 7E and 7F). These results suggested that *nlpD* deletion activated autophagy through AMPK/mTOR pathway modulation.

**Fig 7:**
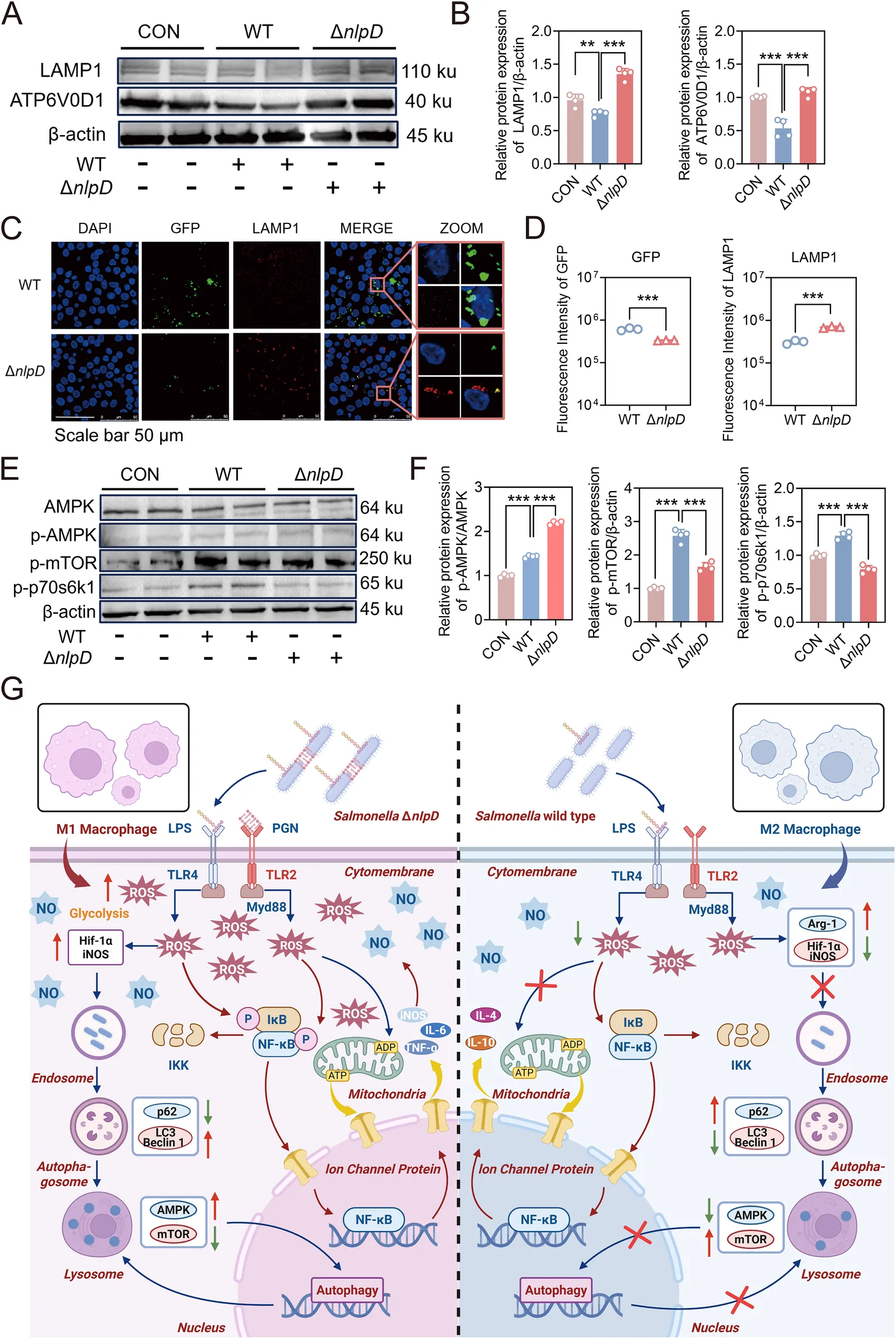
*nlpD* deficiency enhances macrophage lysosomal activity via AMPK–mTOR signaling. **(A)** Protein levels of LAMP1 and ATP6V0D1 of uninfected RAW264.7 (CON) and cells infected by WT strains or Δ*nlpD* mutants of *S.* 4,[5],12:i:- for 6 h (n = 4). **(B)** Quantitative analysis of the gray value of **(A)** using FIJI software. Data were expressed as percentages normalized by CON group. Error bars represent SD; n = 4 independent experiments. Statistical analyses were performed by One-way ANOVA. **P < 0.01; ***P < 0.001. **(C)** Immunofluorescence detection using an anti-LAMP1 (red) in RAW264.7 cells infected with GFP-containing WT or Δ*nlpD* mutants (green) for 6 h. Nuclei were stained with DAPI (blue). Scale bars: 50 μm. **(D)** Quantitative analysis of the fluorescent value revealed that Δ*nlpD* significantly increased LAMP1 fluorescence intensity of cells compared to WT strains. Error bars represent mean ± SEM; data are representative of three independent experiments. Statistical comparisons were performed using Student’s t-test. ***P < 0.001. **(E)** Protein expression (AMPK, p-AMPK, p-mTOR, p-p70s6k1) of uninfected RAW264.7 (CON) and cells infected by WT or Δ*nlpD* mutants for 6 h (n = 4). **(F)** The quantitative analysis of gray value of **(E)** using FIJI software. Data were expressed as percentages normalized by CON group. Error bars represent SD; n = 4 independent experiments. Statistical analyses were performed by One-way ANOVA. **P < 0.01; ***P < 0.001. **(G)** The *nlpD* deletion in *Salmonella* disrupts peptidoglycan remodeling and envelope organization, increasing surface PGN/LPS exposure and enhancing TLR2/TLR4 engagement. This triggers NF-κB signaling, HIF-1α upregulation and a glycolytic shift, as reflected by elevated L-lactate and ATP. These changes promote iNOS/NO production, phagocytosis, and M1 polarization. Concomitantly, Δ*nlpD* infection enhances ROS production and autophagic flux via AMPK-mTOR signaling. Lysosomal activation and autophagosome-lysosome maturation ensure efficient intracellular bacterial clearance through auto-lysosomal degradation.

Elevated ATP6V0D1 and ATP levels in Δ*nlpD*-infected macrophages indicated that these cells are not energy-depleted, apparently contradicting the canonical low-energy paradigm of AMPK activation [40]. Given that glycolytic flux generates ATP while producing AMPK-activating signals, such as mitochondrial ROS and subcellular ATP/ADP gradients, via upstream kinases (LKB1) [41,42], we proposed that AMPK activation here is driven not by energy deficit, but by Δ*nlpD*-induced glycolytic reprogramming. To test whether *nlpD* deletion induces glycolysis, we measured L-lactate levels in RAW264.7 cells infected with WT or *nlpD* mutant strains. Δ*nlpD* infection significantly increased macrophage L-lactate compared with WT (S10A Fig), indicating a pronounced shift toward glycolysis.

To determine whether this glycolytic response accounted for the ATP elevation, we inhibited glycolysis with 2-deoxy-D-glucose (2-DG). In Δ*nlpD*-infected cells, 2-DG reduced L-lactate, whereas in WT-infected cells 2-DG increased L-lactate (S10A Fig). Consistently, 2-DG significantly decreased ATP levels in Δ*nlpD*-infected cells but not in WT-infected cells (S10B Fig), confirming that ATP elevation upon Δ*nlpD* infection is directly fueled by enhanced glycolytic flux. Moreover, 2-DG partially rescued intracellular survival of Δ*nlpD* (S10C Fig), indicating that *nlpD* deletion-induced glycolysis contributes to restricting intracellular *Salmonella* survival. Collectively, these results demonstrate that Δ*nlpD* induces macrophage glycolysis. This metabolic shift increases ATP production and generates AMPK-activating signals, such as mitochondrial ROS (Fig 5H).

Together, *nlpD* deletion in *Salmonella* disrupts peptidoglycan remodeling and envelope organization, increasing surface PGN/LPS exposure and enhancing TLR2/TLR4 engagement. This triggers NF-κB signaling, HIF-1α upregulation and a glycolytic shift, as reflected by elevated L-lactate and ATP. These changes promote iNOS/NO production, phagocytosis, and M1 polarization. Concomitantly, Δ*nlpD* infection enhances ROS production and autophagic flux via AMPK-mTOR signaling. Lysosomal activation and autophagosome-lysosome maturation ensure efficient intracellular bacterial clearance through auto-lysosomal degradation (Fig 7G).

### Screening of anti-*Salmonella* compounds by targeting NlpD protein

Having shown that NlpD is a key regulator of *Salmonella* infectivity and a potential therapeutic target for controlling bacterial infections, we performed structure-based virtual screening of 1,880 FDA-approved drugs against the NlpD protein using Autodock Suite to identify potential anti-*Salmonella* compounds. The drug screening workflow is schematically presented in Fig 8A. Briefly, NlpD protein was used as the receptor and the FDA drug library as ligands. Compounds with a binding affinity ≤ -7 kcal/mol and interactions with ≥ 5 key amino acid residues were prioritized, yielding 8 initial candidates (S3 Table). Next, intracellular proliferation assays showed that ketoconazole (KTZ), obeticholic acid, and pazopanib significantly inhibited replication of the WT strains in macrophages. In contrast, these compounds showed little or no inhibitory effect on the Δ*nlpD* mutants. (S11A Fig). These findings supported an NlpD-dependent anti-*Salmonella* effect. RT-qPCR analysis demonstrated that all three candidate drugs (KTZ, obeticholic acid, and pazopanib) markedly downregulated *nlpD* transcription, with KTZ exhibiting nearly 50% reduction in *nlpD* expression (S11B Fig).

**Fig 8:**
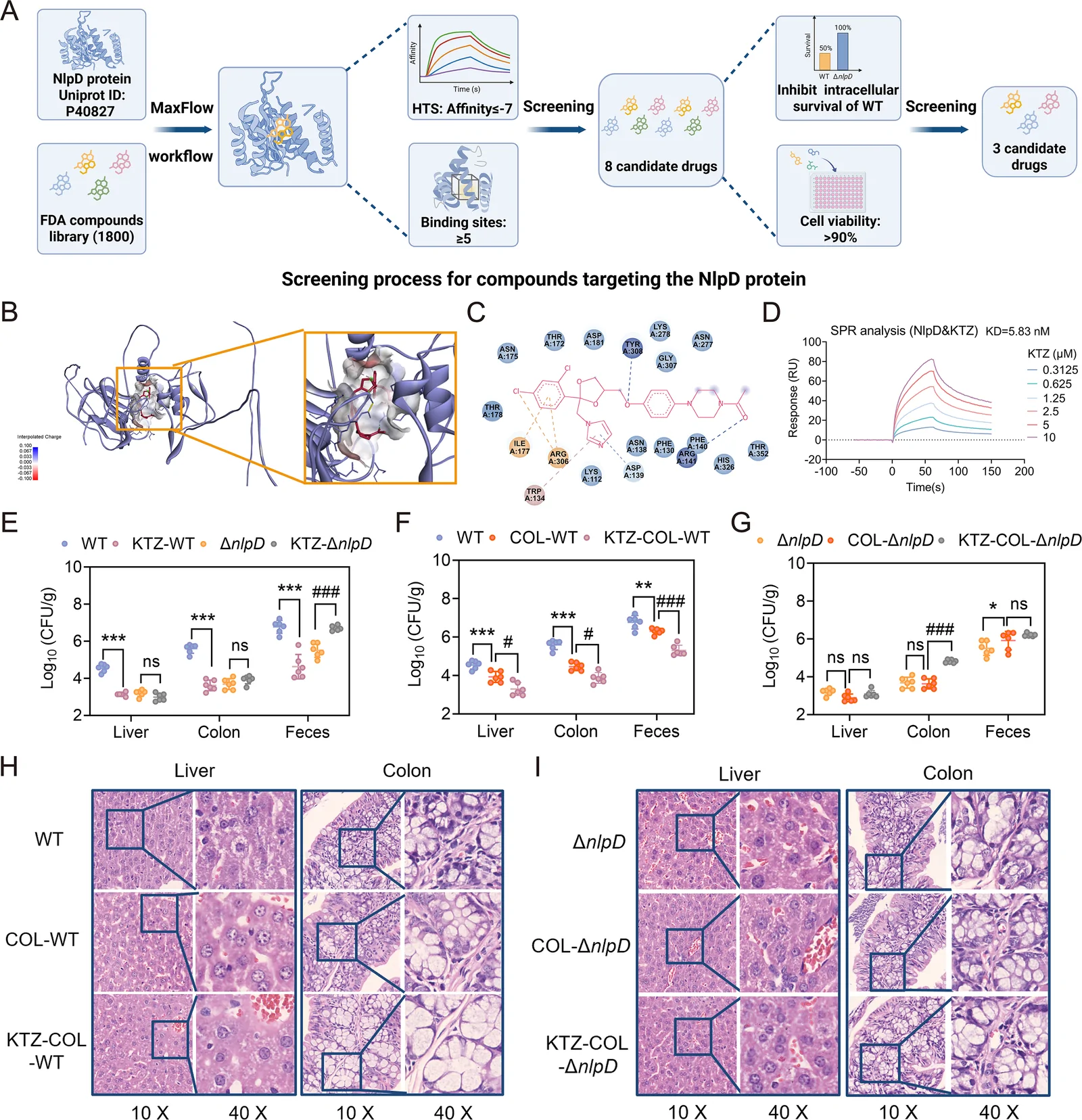
Screening of NlpD-targeted compounds against *Salmonella* infections. **(A)** Schematic overview of the NlpD-targeting ligand screening workflow. **(B)** The binding mode and molecular docking interactions between KTZ and NlpD protein were visualized via Discovery Studio software. KTZ can stably occupy the active binding pocket of the NlpD protein. **(C)** The 2D ligand-receptor interaction diagram. Close-up view highlighting hydrogen bonds between the KTZ and NlpD with key amino acid residues includes ARG141, ASP139, TYR308, TRP134, ARG306, and ILE177. **(D)** Surface plasmon resonance (SPR) analysis showed a robust, dose-dependent binding between KTZ and NlpD, with a calculated KD of 5.83 nM, demonstrating that ketoconazole directly binds to the NlpD protein. **(E)** Bacterial loads of liver, colon, and feces of mice infected with WT or Δ*nlpD* strains (1 × 10^9^ CFU) and treated with or without KTZ (10 mg/kg) for 24 h. Each dot represents an organ from an infected mouse. log_10_ (CFU/g) represents the mean of six mice per group. Error bars represent SD. Data were obtained from two independent experiments. Statistical analyses were performed by Mann-Whitney U test. ***P < 0.001; ^###^*P* < 0.001; ns: not significant. **(F)** Bacterial burden of liver, colon and feces of mice infected with WT strains (1 × 10^9^ CFU) or **(G)** Δ*nlpD* strains (1 × 10^9^ CFU) and treated with PBS (Vehicle), COL (10 mg/kg) and KTZ + COL (5 mg/kg + 5 mg/kg) for 24 h. log_10_ (CFU/g) represents the mean of six mice per group. Error bars represent SD. Data were obtained from two independent experiments. Statistical analyses were performed by Mann-Whitney U test. *P < 0.05; **P < 0.01; ***P < 0.001; ^#^*P* < 0.05; ^###^*P* < 0.001; ns: not significant. **(H)** and **(I)** Representative H&E-stained sections of liver and colon infected by WT or Δ*nlpD* strains treated with of COL (10 mg/kg) or KTZ + COL (5 mg/kg + 5 mg/kg) (10 x and 40 x magnification). H&E staining demonstrated that KTZ treatment significantly attenuated WT strains-induced hepatic injury and colonic inflammation, while showing no therapeutic improvement in Δ*nlpD*-infected tissues.

To further evaluate the therapeutic potential of these candidates against *Salmonella* infection, we first determined their cytotoxicity using CCK-8 assays. The maximum safe concentrations were established as 50 μM for KTZ, 25 μM for obeticholic acid and 25 μM for pazopanib (S11C Fig). Intracellular infection assays with their maximum safe concentrations revealed that both KTZ and obeticholic acid consistently inhibited intracellular WT strains replication but exerted no significant bactericidal effects against Δ*nlpD* strains. In contrast, pazopanib showed bactericidal activity against both WT and Δ*nlpD* strains (S11D Fig). The inhibitory effect of KTZ on intracellular survival of a clinical strain *S*. Pullorum WF770 in HD11 cells was also abolished in Δ*nlpD* (S11E Fig). Among the tested candidates, KTZ exhibited the highest safety in CCK8 assays, while obeticholic acid showed weak and unstable activity. Pazopanib was excluded due to its non-specific bactericidal activity. As a result, KTZ was prioritized as a repurposing candidate for further antibacterial development. Further molecular docking analysis demonstrated high-affinity binding of KTZ to NlpD protein with ΔG of -9.07 kcal/mol and interactions spanning seven critical residues, including ARG141, ASP139, TYR308, TRP134, ARG306, and ILE177 (Fig 8B and 8C; S3 Table). To demonstrate direct binding between KTZ and NlpD, we performed surface plasmon resonance (SPR) analysis using purified NlpD protein (S12A Fig). SPR analysis demonstrated specific binding with a dissociation constant (KD) of 5.83 × 10^-6^ M, confirming direct KTZ-NlpD interaction (Fig 8D). These results demonstrate that KTZ effectively impairs the intracellular *Salmonella* survival in cellular models by directly targeting NlpD and suppressing its expression.

Furthermore, we evaluated KTZ’s therapeutic potential in a murine *Salmonella* infection model. Consequently, KTZ treatment significantly reduced bacterial burdens in systemic (liver) and intestinal (colon, feces) compartments of mice infected with WT strains by 1- to 2-log_10_ while exhibiting no efficacy against the Δ*nlpD* mutants, confirming NlpD-dependent ant-infective ability *in vivo* (Fig 8E). Notably, KTZ exhibited marked synergy with colistin in WT-infected mice. Compared with colistin monotherapy, the combination treatment resulted in an additional 1-1.5 log_10_ reduction in bacterial burdens in the liver, colon, and feces (Fig 7F). By contrast, this combinatorial effect was entirely abrogated in Δ*nlpD*-infected mice (Fig 8G), confirming the NlpD-dependence of KTZ’s potentiation mechanism. Consistently, checkerboard assays revealed synergistic activity between KTZ and colistin against WT *in vitro*, with a fractional inhibitory concentration index (FICI) of 0.5 (S12B Fig). Meanwhile, time-kill curves showed that KTZ markedly enhanced the antibacterial activity of colistin. In contrast to the progressive bacterial growth observed with individual treatments, the KTZ-colistin combination suppressed bacteria within 6 h and lowered viable counts to 4.5 log_10_ CFU/mL at 12 h, corresponding to an approximately 3 log_10_ CFU/mL reduction relative to the single agent (S12C Fig). H&E staining demonstrated that KTZ treatment significantly attenuated WT strains-induced hepatic injury and colonic inflammation, while showing no therapeutic improvement in Δ*nlpD*-infected tissues (Fig 8H and 8I). Collectively, these results demonstrate that KTZ exerts potent anti-*Salmonella* effects through specific targeting NlpD, positioning it as a promising therapeutic candidate for combating *Salmonella* infections.

### ketoconazole displays favorable safety profiles in mice

Next, we fully evaluated the safety profiles of KTZ at given dose in a murine model [43]. Simply, we intraperitoneally administered 30 mg/kg KTZ to 7-week-old male BALB/c mice, using DMSO as the vehicle control. Mice were euthanized at 2- and 14-day intervals post-administration. Blood samples were collected for comprehensive analysis, including ALT, AST for liver, UREA, CREA for kidney function, and Na^+^, K^+^, Cl^−^ for ion homeostasis. Liver and kidney were harvested for H&E histological analysis at 2- and 14-day post-administration, respectively (Fig 9A). Notably, KTZ did not affect the survival, body weight (Fig 9B and 9C) and blood biochemical indices in mice. Normal ALT/AST and UREA/CREA levels indicated no hepatic or renal toxicity (Fig 9D). Stable serum concentrations levels of Na^+^, K^+^, and Cl^−^ in KTZ-treated mice confirmed maintenance of electrolyte homeostasis (Fig 9E). Meanwhile, H&E staining results revealed no significant pathological alterations in KTZ-treated mice compared to vehicle controls (Fig 9F). These findings indicate that KTZ did not induce detectable histopathological damage in the liver or kidney under the tested conditions. Additionally, KTZ exhibited exceptional blood compatibility, demonstrating < 8% hemolysis in mammalian RBCs at 256 μg/mL (Fig 9G), confirming that KTZ had low toxicity to mammalian cells. These results prove that KTZ emerges as a mechanistically distinct anti-*Salmonella* agent with great safety profiles.

**Fig 9:**
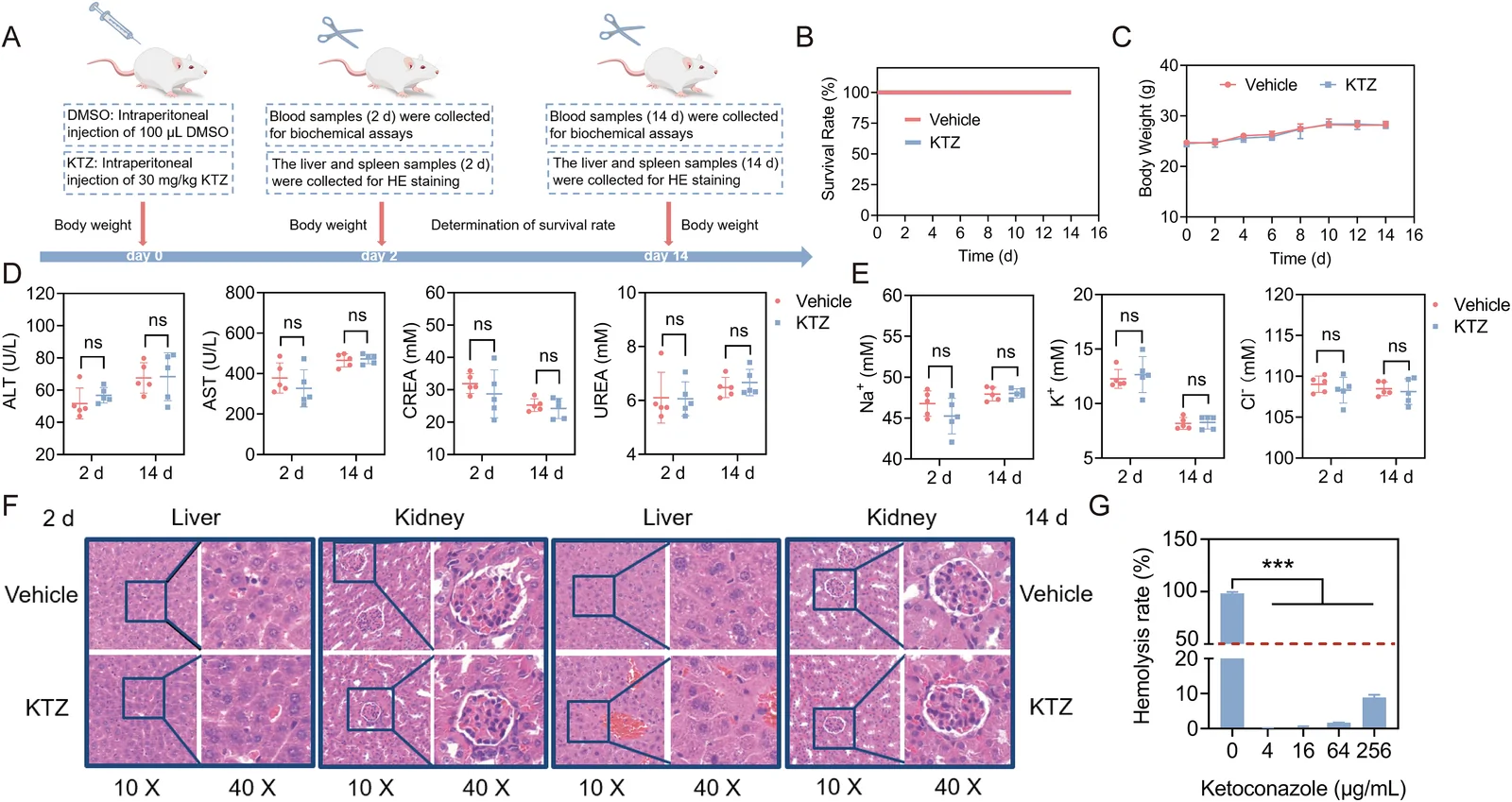
Ketoconazole demonstrates favorable safety profiles in mice. **(A)** Schematic illustration of safety evaluation of KTZ in mice. Mice were intraperitoneal (*i.p.*) treated with vehicle (DMSO) or KTZ (30 mg/kg) for 14 days (n = 5). **(B)** Survival curves and **(C)** body weight of mice treated with vehicle or KTZ (30 mg/kg) for 14 days (n = 5). KTZ showed no effect on survival and body weight. **(D)** Blood biochemical indices of ALT (alanine aminotransferase), AST (aspartate aminotransferase), UREA and CREA (creatinine) of the mice after treatment with vehicle or KTZ at day 2 and day 14. Error bars represent SD; n = 5 independent experiments. Statistical analyses were performed by Two-way ANOVA. ns: not significant. **(E)** Serum electrolyte profiles of K^+^, Na^+^ and Cl^-^ after treatment with vehicle or KTZ at day 2 and day 14. Error bars represent SD; n = 5 independent experiments. Statistical analyses were performed by Two-way ANOVA. ns: not significant. **(F)** H&E staining of liver and kidney sections in vehicle or KTZ-treated (30 mg/kg) mice at either day 2 or day 14. KTZ exhibited no significant pathological alterations in liver and kidney compared to vehicle controls. **(G)** Hemolytic activity of varying concentrations of KTZ toward mammalian red blood cells (RBCs), while sterile water and PBS were used as positive or negative controls, respectively. KTZ exhibited exceptional blood compatibility. Error bars represent SD; n = 3 independent experiments. Statistical analyses were performed by One-way ANOVA. ***P < 0.001.

## Discussion

The monophasic variant 4,[5],12:i:- (*fljB*-deficient) has emerged as a major global pathogen since the early 2000s due to its enhanced epidemiological fitness [2,44]. Analysis of 13,612 genome sequences from the NCBI Pathogen Detection database revealed that 65% of strains exhibited multidrug resistance (MDR), this serovar as the most prevalent MDR *Salmonella* [45]. Notably, 10% of isolates are quinolone-resistant and 2% colistin-resistant, with 60% of the latter concurrently harboring quinolone resistance genes [45]. This emerging resistance to both last-resort colistin and first-line fluoroquinolones [46,47], severely compromising treatment options for life-threatening infections. In this study, we identified *nlpD* as a critical virulence determinant for intracellular persistence of *S*. 4,[5],12:i:-, elucidating its mechanism through TLR-mediated NF-κB activation, M1 macrophage polarization and autophagic clearance. From a One Health perspective, targeting virulence and fitness determinants such as NlpD offers a viable alternative to conventional antibiotics. In livestock, this reduces antimicrobial use while curbing multidrug-resistant bacteria, aligning with national AMR action plans that prioritize reducing veterinary antibiotics and fostering innovative disease management.

Although KTZ is canonically used as an antifungal targeting fungal CYP51 [48], accumulating evidence indicates that azoles possess broader antimicrobial potential [49]. Moreover, azole-based combinations also exhibit synergistic effects with other agents, underscoring their repurposing value [50,51]. Nevertheless, whether azole drugs can be repurposed to target bacterial virulence-associated determinants remains unexplored. Here, we identify KTZ as a potent anti-*Salmonella* compound that targets the cell-envelope regulator NlpD, revealing an unexpected link between antifungal repurposing and bacterial envelope homeostasis. Clinically, systemic KTZ administration carries hepatotoxicity risk, limiting its utility in chronic regimens [52,53]. Although no overt toxicity was observed in mice treated with KTZ (30 mg/kg) for 14 days, this liability warrants careful consideration for translational development.

High-throughput transposon insertion sequencing methods, such as TraDIS, have transformed functional genomics in understudied bacteria by enabling genome-wide identification of genotype-phenotype relationships [54]. TraDIS integrates phenotypic fitness profiling with next-generation sequencing to assess the contribution of all bacterial genes to survival under selective pressures, making it powerful for identifying genes associated with bacterial adaptation or antibiotic resistance. For example, TraDIS screening in a mouse model revealed 15 bacterial fitness determinants, with Δ*fimG* showing enhanced *in vivo* persistence despite defective epithelial adhesion [55]. TraDIS was also used to identify genes enabling UPEC to evade TLR-induced zinc toxicity, with mutants maintaining normal intramacrophage survival through evasion mechanisms [56]. Using TraDIS, we identified *nlpD* as a virulence-associated gene critical for *Salmonella*’s intracellular survival. Notably, although Tn-seq screens identify bacterial virulence determinants, they inherently overlook host factors; complementary CRISPR-Cas9 screening will contribute to elucidate host-pathogen interplay during *Salmonella* infection [57].

NlpD is a bacterial peptidoglycan hydrolase activator essential for cell division [13]. Here, we demonstrated that *nlpD* deletion in *Salmonella* enhanced the exposure of peptidoglycan and LPS, increasing susceptibility to oxidative stress and macrophage killing, thereby compromising intracellular survival. Although NlpD regulates *rpoS* in *E. coli* [58], this regulatory link is minimal in *S*. Dublin due to promoter divergence [15], and *nlpD* deletion impairs survival specifically under combined acid-oxidative stress rather than acid alone [59], indicating *rpoS*-independent virulence functions. Mechanistically, NlpD orchestrates M2 macrophage polarization and suppresses autophagy to enhance infectivity, establishing it as a multifunctional virulence factor beyond conventional prediction. Notably, while recombinant NlpD suppresses inflammation in uropathogenic *E. coli* infection [60,61], our study reveals that *nlpD* deletion instead enhances host TLR-NF-κB signaling and autophagic flux, enabling effective bacterial clearance before systemic hyperinflammation develops. This early control prevents the maladaptive immune activation characteristic of severe salmonellosis. These findings position NlpD as a critical molecular rheostat that fine-tunes the balance between protective immunity and immunopathology, offering a promising target against systemic *Salmonella* and other infections.

The NF-κB complex regulates immune responses and maintains host physiology [62]. During infection, *Salmonella* activates the NF-κB pathway, triggering the release of inflammatory cytokines like IL-6 and IL-1β to aid bacterial clearance [63]. However, *Salmonella* has evolved strategies to evade host immunity by suppressing NF-κB signaling. For instance, SPI-2 effectors SseK1 and SseK3 inhibit IκB degradation in macrophages, dampening NF-κB activation [64]. Similarly, the T3SS effector SpvD blocks p65 nuclear import, suppressing NF-κB-dependent transcription and enhancing bacterial dissemination by weakening host defenses [65]. In our study, *nlpD* deletion in *Salmonella* increased LPS/peptidoglycan content, boosting TLR4 and TLR2 expression and subsequently upregulating the NF-κB pathway. This led to higher levels of pro-inflammatory cytokines (e.g., IL-6, TNF-α), which were detrimental to *Salmonella* survival. These findings suggest that *nlpD* is crucial for *Salmonella*’s anti-inflammatory strategy and intracellular survival.

*Salmonella* infection induces macrophage differentiation into M1 (pro-inflammatory) or M2 (anti-inflammatory) phenotypes, depending on the cytokines present [66]. M1 macrophages, stimulated by IFN-γ or LPS via TLR4, secrete pro-inflammatory cytokines and kill bacteria. In contrast, M2 macrophages, driven by IL-4, secrete anti-inflammatory cytokines, resolve inflammation, and promote healing [67]. It has been showed that *Salmonella* favors M2 macrophages during chronic infection, likely to enhance its intracellular survival [68]. Generally, impaired autophagy typically shifts macrophage polarization toward M1, increasing inflammation. A notable example is ezetimibe, which alleviates steatohepatitis and reduces inflammation by inducing mTOR-independent autophagy activation, thereby favoring M2 macrophage polarization [69]. Similarly, low fluoride levels activate the PINK1/Parkin-mediated mitophagy pathway, triggering systemic inflammation and promoting macrophage polarization toward the M2 phenotype [70]. Conversely, activating autophagy and NF-κB signaling can convert M2-like macrophages to M1, enhancing bacterial clearance [71]. RP-182, a synthetic peptide, reprograms M2 macrophages to M1 by activating autophagy and phagocytosis, increasing pro-inflammatory cytokines like TNF-α [72]. In HSV-1 infections, M1 macrophages suppress viral replication through autophagy, highlighting their role in pathogen control [73]. In line with these findings, our study uncovered that *nlpD* deletion markedly alters host macrophage polarization, reprogramming the bacteria-permissive M2 phenotype into a bactericidal M1-dominant state. This immunomodulatory shift is accompanied by significantly enhanced autophagic flux, and collectively reducing intracellular *Salmonella* survival.

The bacterial cell envelope is a tightly integrated system, and genetic perturbation of septal PGN remodeling produces pleiotropic effects on outer-membrane architecture and host recognition. Although NlpD deletion causes cell-division defects, the precise biochemical link between septal PGN defects and LPS homeostasis remains elusive. We proposed that impaired septal remodeling compromises envelope integrity through disrupted outer membrane-PGN coupling, altered microdomain organization, and enhanced outer-membrane vesicle release; however, these mechanisms remain speculative. Biochemical characterization of peptidoglycan turnover and muropeptide composition is required to determine whether NlpD loss increases surface accessibility or release of immunostimulatory cell-wall components.

In conclusions, our results establish NlpD as a critical virulence factor enabling *Salmonella* immune evasion and intracellular persistence. Mechanistically, NlpD deletion reprograms macrophages from M2 to M1 polarization, enhancing autophagy and bacterial clearance. We further demonstrate that the FDA-approved antifungal KTZ exhibits potent anti-*Salmonella* activity through specific NlpD targeting. However, clinical translation is constrained by hepatotoxicity and unfavorable pharmacokinetics [52,74], positioning KTZ as a lead scaffold for optimized analogues rather than a standalone therapeutic. Future efforts should prioritize structure-based design of NlpD inhibitors with improved safety profiles and sustained efficacy, while long-term resistance stability requires rigorous evaluation. Collectively, our work validates NlpD as a druggable target for anti-salmonellosis therapy and provides a mechanistic framework for future intervention strategies.

### Limitations

Despite the mechanistic insights provided by this study, several inherent limitations warrant consideration. Firstly, while key findings were validated in BMDMs, much of the initial mechanistic work relied on immortalized cell lines, which may not fully replicate the physiological complexities *in vivo*. Secondly, the murine infection model employed here primarily represents an acute infection paradigm and may not fully reflect long-term carriage or chronic persistence of *Salmonella* 4,[5],12:i:- in humans or food-producing animals. Future investigations using chronic infection or avian models will therefore be valuable to delineate the contribution of NlpD to long-term bacterial persistence. Thirdly, although ketoconazole exhibited antibacterial activity and reduced bacterial burden *in vivo*, the current study evaluated only a single treatment regimen. Comprehensive pharmacokinetic and pharmacodynamic analyses, coupled with dose-optimization studies, are required to fully evaluate its therapeutic potential in bacterial infections. Furthermore, since KTZ can modulate host cytochrome P450 enzymes, potential host-mediated or off-target effects cannot be completely excluded and should be addressed in future work. Finally, although NlpD was identified as an essential virulence determinant in *Salmonella* 4,[5],12:i:-, whether this mechanism is conserved among other *Salmonella* serovars and diverse clinical isolates remains to be determined. Validation across an expanded strain panel will be essential to establish the broader relevance of NlpD as a target for controlling *Salmonella* infections.

## Materials and methods

### Ethical statement

This study was conducted according to the relevant guidelines of Jiangsu Laboratory Animal Welfare and Ethical of Jiangsu Administrative Committee of Laboratory Animals (SYXK-2022–0044). All animal experiments were approved by the Animal Care Committee of Yangzhou University. The laboratory animal usage license number is SCXK-2022–0009, certified by the Jiangsu Association for Science and Technology.

### Mice

BALB/c mice were purchased from the Institute of Comparative Medicine at Yangzhou University. Male mice aged 6–8 weeks were used. Mice were housed under specific pathogen-free conditions with six mice per cage. All mice were raised in an environment with a strict 12-hour light cycle, standard room temperature (21-26 °C) and relative humidity (50-60%). And the mice were supplied with autoclaved double-distilled water (ddH_2_O) and Mouse-Breeding complete feed for mice *ad libitum*. The mice acclimated to the facility before experiments. All mice and rats were euthanized by cervical dislocation.

### Bacterial strains and culture medium

*S.* 4,[5],12:i-, *S.* Typhimurium strain 14028S and *S.* Pullorum WF770 were used in this study. *S.* 4,[5],12:i- harbored GFP on plasmid pXG-10SF was used for confocal microscopy in this study. pXG-10SF was kindly provided by Professor Guoqiang Zhu (College of Veterinary Medicine, Yangzhou University, Yangzhou, China). Bacteria for late-log-phase infections were grown in LB overnight at 37 °C (200 rpm), then diluted into complete media (DMEM with 10% FBS) at MOI of 10 for macrophage infection.

### Cell culture and culture medium

RAW264.7 cells were obtained from Cyagen Biosciences (Guangzhou, China), while chicken macrophage HD 11 cells were purchased from Yaji Mall (Shanghai, China). Both cell lines were cultivated in high-glucose DMEM (Gibco, New York, USA) supplemented with 10% fetal bovine serum (FBS) in an atmosphere of 5% CO_2_ at 37 °C. Experiments were performed with RAW264.7 cells between passages 3 and 16.

Bone marrow cells were aseptically isolated from the femurs and tibias of C57BL/6 mice. The cell pellet was resuspended in complete DMEM supplemented with 10% fetal bovine serum (FBS) and 20 ng/mL recombinant mouse macrophage colony stimulating factor (M-CSF, PeproTech, USA). Cells were maintained at 37 °C with 5% CO_2_. Fresh medium containing FBS and M-CSF was replaced on day 5. By day 7, adherent cells exhibiting typical macrophage morphology were considered fully differentiated bone marrow-derived macrophages (BMDMs) and were used for subsequent experiments.

### *Salmonella* infection model

Murine macrophage cells RAW264.7 were cultured in DMEM containing 10% FBS at 37 °C in 5% CO_2_. Overnight-grown bacteria (WT and Δ*nlpD*) were incubated on monolayer cells at a multiplicity of infection (MOI) of 10 in 24-well plates at 37 °C for 1 h. The cells were gently washed twice with PBS solution after 1 hpi. The 200 µg/mL kanamycin diluted with DMEM was incubated on infected cells to kill extracellular bacteria. After 0.5 hpi, the infected cells were washed twice with PBS to remove loosely adhering bacteria. Infections were done in duplicate. One set: cells lysed with 0.1% Triton X-100 for 30 min, lysates diluted and plated on XLD agar to calculate original engulfed bacteria (phagocytosis resistance ratio). The other: after 6 h incubation, cells were washed, lysed and plated onto XLD agar to count surviving bacteria (intracellular survival ratio).

Six-week-old BALB/c mice (n = 6 per group, 20 ± 2 g) were inoculated by oral administration with 10^9^ CFU WT or Δ*nlpD* strains. After 24 hpi, mice were euthanized by cervical dislocation. The liver, spleen, colon, cecum and feces were collected and serially diluted 10-fold with PBS, then plated on XLD agar plates to count CFU. For the drug-treated group, mice were intraperitoneally treated with vehicle (DMSO), 10 mg/kg of COL, 10 mg/kg of KTZ or 10 mg/kg of KTZ and COL at 2 hpi of WT and Δ*nlpD* strains, respectively. Animals were euthanized by cervical dislocation after 24 h with treatment of drugs. Livers colon and faeces were collected and homogenized to 1 mL of PBS, then ten-times dilution series for plating onto XLD agar plates to enumerate the CFU.

### Cell viability assay

Cell viability was measured with the Cell Counting Kit-8 (Beyotime, Nantong, China) assay. Briefly, cells were seeded into 96-well plates and incubated for 12 h in a 5% CO_2_ atmosphere at 37 °C. Following 12 h of drug treatment (with 0.5% DMSO as a control), 10 μL of CCK-8 solution was added to each well, and the plates were incubated at 37 °C for 2 h. Absorbance at 570 nm of cells was measured by a microplate reader.

### Construction of the transposon random mutant library

Tn5 insertion mutants of *S.* 4,[5],12:i- (*mcr-3*) were constructed using the pUTmini-Tn5km2 plasmid (Ampᵣ Kmᵣ). The plasmid was purified from bacterial clones, transformed into donor *E. coli* X7213, and conjugated with *S.* 4,[5],12:i- as recipient. Donor and recipient strains were grown to an OD_600_ of 1.0, harvested cultures and washed with PBS, resuspended in LB broth, then mixed and incubated at 37 °C for 6 h on LB agar with 2,6-diaminoheptanedioic acid (DAP). Cells were plated on 10 LB agar plates supplemented with kanamycin and incubated overnight. Total counts were estimated via colony counts across replicates. Repeating this 30 times yielded a Tn5 random mutant library with 100,000 mutants (input library), established for subsequent analysis [75].

### *Salmonella* infection-essential genes selection on the library

The transposon random mutant library was cultured in LB broth with kanamycin and colistin for 6 h. After washed with PBS, part was adjusted to an OD_600_ of 1.0 as an input library. Another part infected RAW264.7 cells for 6 h. And the suspension was plated on kanamycin-LB agar plates, with colonies harvested next day as an output library. Tn5 identification primers were to verified kanamycin resistance gene to ensure its purity. DNA was extracted using TIANamp kit (TianGen) per instructions. TraDIS-specific sequencing on HiSeq 2500 with 50-bp single-end reads generated transposon-directed reads as described.

To identify random transposon insertion sites in mutant genomes, a TraDIS library was prepared with Tn5-specific adapters. This method enriches genuine transposon-chromosome junctions by blocking reverse primer hybridization, ensuring reads start with 10 bp of transposon sequence followed by downstream genomic sequence. Reads were mapped via SMALT (Wellcome Trust Sanger Institute). Log_2_ fold change (Log_2_FC) quantified read number differences between control and selection groups relative to input, using ≥ 2 and ≤ -2 as cutoffs to identify genes with significant insertion variations.

Genes essential for intracellular survival were defined according to the following criteria: (i) an insertion count of ≤1 in the output library, (ii) at least one insertion detected in the input library, and (iii) a significant difference in representation between the input and output libraries (*P* < 0.05). Genes showing higher insertion in the input library but marked depletion or complete loss in the output library were considered to have a greater contribution to intracellular fitness and were prioritized for experimental validation.

### Mutant construction

Mutants were constructed using the suicide vector pRE112. All oligonucleotide primers used in this study were purchased from GENEWIZ (Nanjing, China). Mutants were created using *S*. 4,[5],12:i-, *S.* Typhimurium 14028S and *S.* Pullorum WF770. The primers used to construct the mutations were as follows in S4 Table, and the mutations confirmed by PCR and DNA sequencing.

### Minimum inhibitory concentration (MIC) determination

MICs of drug were determined via serial 2-fold dilutions in Mueller-Hinton Broth (MHB) following the CLSI 2021 guidelines. Simply, bacterial cultures were diluted 1:1000 in MHB to a final concentration of 1.5 × 10^6^ CFUs/mL. Equal volumes of this bacterial suspension and drug solutions were mixed in 96-well microtiter plates. After incubation at 37 °C for 16–18 h, the MIC was defined as the lowest drug concentration at which no visible bacterial growth was observed.

### Transcriptomics analysis

Samples were divided into four groups for transcriptome sequencing [76]. “WT” and “Δ*nlpD*” groups: overnight strains inoculated 1:100 in LB, cultured at 37 °C (200 r/min) for 6 h to OD_600_ = 0.5. “WT-cell” and “Δ*nlpD*-cell” groups: 6-well macrophages infected with respective overnight strains at a MOI = 10, bacterial cells were collected 6 hpi. All groups were washed with PBS and harvested with 1 mL TRNZOL for analysis.

RNA sequencing was performed using Hiseq 2000 (Illumina, San Diego, America) with 150 bp paired-end modules. Raw sequencing reads were filtered by quality control and mapped against the genome of 14028S/RAW264.7. Further analyses were conducted and sequenced on an Illunima Novaseq 6000 sequencer by Majorbio Company (Shanghai, China). Differentially expressed genes were identified by gene expression-level analysis using the FPKM (Fragments Per Kilobase of transcript per Million mapped reads) method with P-values ≤ 0.05 and fold change (FC) values ≥ 2 (log_2_ FC ≥ 1 or log_2_ FC ≤ -1).

### Time-dependent killing curves

Overnight bacterial cultures were diluted 1:1000 in MHB. Bacteria were then exposed to different concentrations of H_2_O_2_ (0, 2.5 and 3 mM) under different pH (4, 5, and 7) environment. At each time point (0, 6, 12 and 24 h), samples were subjected to ten-fold serial dilution and plated on MHA plates. Following overnight incubation, CFUs were enumerated to calculate.

### SOD activity determination

Overnight cultures of *S.* 4,[5],12:i:- were diluted at 1:100 in fresh medium and incubated for 6 h. Bacterial suspensions were adjusted to an OD_600_ of 0.5 using PBS. The superoxide dismutase (SOD) activity of *S.* 4,[5],12:i:- was measured using the Total Superoxide Dismutase Assay Kit with WST-8 (Beyotime, Shanghai).

### Intracellular ATP assay

The ATP levels of *S.* 4,[5],12:i:- or *Salmonella*-infected RAW264.7 cells were measured using an Enhanced ATP Assay Kit (Beyotime, Shanghai). Treated cells were centrifuged at 12,000 rpm at 4 °C for 7 min, and the supernatant was collected. Subsequently, 100 μl of ATP detection working solution was added into a 96-well plate and incubated for 5 min at room temperature, followed by 20 μl sample addition and mixing. Luminescence was quantified after 5 s using a Infinite E Plex Microplate reader (TECAN). ATP concentrations were calculated by referencing the standard curves.

### Scanning electron microscope (SEM)

The surface morphology alterations of WT or Δ*nlpD* strains of *S.* 4,[5],12:i:- were evaluated by scanning electron microscope (SEM). Briefly, overnight cultures were diluted at 1:100 in fresh medium and incubated for 6 h. Then bacterial cells were pelleted by centrifugation, washed twice in PBS, and fixed overnight at 4 °C using 2.5% glutaraldehyde. Next, samples were dehydrated by gradient ethanol (30%-100%). The specimens were then dried, gold-coated. And imaging was performed using GeminiSEM 300 (ZEISS, Germany).

### Cell membrane permeability assay

Propidium iodide (PI) and N-phenyl-1-naphthylamine (NPN) were used to assess cell membrane permeability. Briefly, overnight bacteria (1:100 dilution) cultured 6 h to OD_600_ = 0.5, incubated with PI (5 μM) or NPN (10 μM) at 37 °C for 30 min. Mixtures in 96-well plates were tested for fluorescence via Tecan Infinite M200 (PI: 535/615 nm; NPN: 350/420 nm).

### Cell membrane fluidity assay

Laurdan was used to detect cell membrane fluidity. Bacteria incubated with 10 μM Laurdan for 30 min and added to 96-well plates. Tecan Infinite M200 was used to measure fluorescence (excitation 350 nm; emission 435/490 nm). The Laurdan GP was calculated with the formula of GP = (I_435_-I_490_)/ (I_435_ + I_490_). All experiments were conducted in biological triplicate.

### Reactive oxygen species (ROS) detection

Infected/uninfected RAW264.7 cells were seeded in 96-well plates for 6 h. Intracellular ROS was detected using 10 μM 2′,7′-dichlorodihydro-fluorescein diacetate (DCFH-DA) (Beyotime, China). Cells incubated with DCFH-DA at 37 °C for 30 min after washed twice with PBS. Fluorescence intensity (488/525 nm) was measured via a microplate reader (BioTek Synergy 4, USA).

### Nitric oxide (NO) assay

Nitric oxide (NO) was measured via Griess staining. After infection of RAW264.7 cells by WT or Δ*nlpD* strains for 6 h, the supernatants were collected for subsequent assays. 50 μL supernatant, 50 μL Griess Reagent I and 50 μL Reagent II were added successively to 96-well plates. Meanwhile, 50 µL standard sodium nitrate solution at concentrations of 0, 1, 2, 5, 10, 20, 40, 60 and 100 µM was prepared to generate a standard curve. Samples and standards were read at 540 nm via an ELISA reader (Stat Fax 2100, USA).

### Enzyme-linked immunosorbent assay (ELISA)

Mice organs: 1 g liver/spleen/colon/cecum were diluted with 100 μL PBS, centrifuged (12000 r/min, 10 min), and supernatants stored at -20 °C. Inflammatory cytokines (IL-1β, IL-6, TNF-α, IL-4, IL-10) were analyzed via ELISA kits (Shanghai Enzyme-linked Biotechnology Co., Ltd. Shanghai, China). Simply, 50 μL samples/standards were added to 96-well plates, followed by 50 μL antibody-enzyme complex. After washing five times, 50 μL chromogenic solution was added to each well and incubated at 37 °C for 10 min. 50 μL termination solution was added to stop the assay. Absorbance at 450 nm was measured and results calculated using a standard curve.

Infected cells: WT or Δ*nlpD* strains-infected cells were harvested by centrifugation at 3000 r/min for 10 min, and cell supernatants were collected about 500 μL for examination of inflammatory cytokines (IL-1β, IL-6, TNF-α, IL-4 and IL-10).

Bacteria samples: overnight WT and Δ*nlpD* strains were inoculated 1:100 in LB, cultured at 37 °C for 6 h to OD_600_ = 0.5. The contents of Lipopolysaccharide (LPS), Peptidoglycan (PGN), Phosphatidylglycerol (PG) and Phosphatidylethanolamine (PE) were tested using respective Elisa kits, following the above-described methods.

### RNA extraction and RT-qPCR analysis

Overnight WT or Δ*nlpD* strains were inoculated 1:100 in LB, cultured at 37 °C (200 r/min) for 6 h to OD_600_ = 0.5. Bacteria washed twice with PBS and 500 μL RNA-easy reagent added. Total RNA was extracted using the RNA-easy isolation reagent (Vazyme, Nanjing, China) following the instructions provided and quantified via Nanodrop spectrophotometer (Thermo Scientific) of absorbance 260/280 nm. 1 μg RNA reverse-transcribed using the HiScript III RT SuperMix for qPCR (+gDNA wiper) Kits. RT-qPCR assay was performed by 7500 Fast Real-Time PCR System (Applied Biosystem, CA, USA) using the ChamQ Universal SYBR Color qPCR Master Mix Kitswith the optimized primers. Target genes’ relative expression 2^−ΔΔCT^ was normalized to *16S*. The primer sequences were presented in S5 Table.

### Immunoblotting analysis

Protein extraction pre-treatment followed the above method. Wells washed twice with PBS and collected in 1.5 mL tubes for the following centrifuged (1000 r/min, 5 min, 4 °C). Precipitate was resuspended in 200 μL RIPA (Beyotime, Nantong, China) (with 1 mM PMSF (Beyotime, Nantong, China) and 1 mM phosphatase inhibitors). Put samples into ice for 20 min to extract protein after a 1 mL syringe breaking up cells by repeating suction. Cells were centrifuged at 10000 r/min in a 4 °C centrifuge for 7 min. Supernatants (proteins) stored at -80 °C for subsequent experiments.

For immunoblotting, proteins mixed with 5 × loading buffer were boiled at 100 °C for 10 min and stored at -80 °C. Equal proteins were separated by SDS-PAGE and transferred to polyvinylidene difluoride membrane (PVDF) (Sigma). Blots were probed with primary antibodies (S6 Table) at 4 °C overnight and HRP-goat anti-rabbit secondary at room temperature for 2 h. Signals detected via Super Signal West Pico chemiluminescence substrate (Thermo), quantified by ImageJ software (GE Healthcare Life Sciences, UK).

### Immunofluorescence analysis

RAW264.7 cells were incubated on slides in 12-well plates for 12 h. Overnight WT or Δ*nlpD* strains were adjusted OD_600_ = 0.25 to infect cells for 6 h at MOI = 10. After fixation with 4% paraformaldehyde (Beyotime, Nantong, China) for 30 min, cells were washed with PBS and permeabilized with 0.1% Triton X-100 for 30 min at room temperature. After blocking for 10 min, cells were incubated with primary antibodies (1:200) overnight at 4 °C. Following PBS washes, goat anti-rabbit HRP secondary antibodies (1:50) were added at 37 °C for 1 h. Nuclei were stained with 5 μg/mL DAPI staining for 20 min. Slides mounted with 90% glycerol were examined via Leica TCS SP2 confocal microscope.

### Intracellular L-lactate measurement

Intracellular L-lactate levels in RAW264.7 macrophages were quantified with a commercial L-lactate assay kit (Elabscience, China). Following infected with WT or Δ*nlpD* strains for 6 h, macrophages were washed with cold PBS and lysed. The lysates were centrifuged at 12,000 × g for 10 min at 4 °C to collect the supernatant. After adding the working reagent, samples were incubated at 37 °C followed by absorbance measurement at 530 nm using a microplate reader. L-lactate concentrations were calculated from the standard curve and normalized relative to the total protein content, which was determined by the BCA protein assay.

### siRNA-directed gene knockdown

The siRNA knockdown was performed as previous studies [43]. RAW264.7 cells were seeded in 6-well plates at a density of 1 × 10^6^ cells per well before transfection to reached 50%-70% confluence. Prior to transfection, 250 μL Opti-MEM reduced-serum medium was used to dilute 0.5 μg siRNA (sequences in S2 Table; AZENTA Life Science, Suzhou, China). 250 μL Opti-MEM was also used to dilute 6 μL Lipofectamine 2000 (Invitrogen, USA). The diluted siRNA and Lipofectamin 2000 were mixed and incubated for 20 min at room temperature to form complexes. Then culture removed medium from the cells. The siRNA- Lipofectamin 2000 complexes were added dropwise to each well. After 6 h of incubation, the transfection medium was then replaced with fresh complete medium. Cells were collected at 24 h and 48 h after transfection to assess the best knockdown efficiency. Western blot with an anti-Beclin 1 antibody was used to confirm Beclin 1 knockdown.

### Virtual screening of inhibitors targeting NlpD protein

The molecular docking was based on the protein-ligand docking using MaxFlow for virtual screening. Screening workflow concludes the preparation of receptors and ligands structures, docking and scoring. The receptor was NlpD protein and the ligands were derived from FDA compounds library (1800 compounds). During receptor structure preparation, the bound ligand and water molecules were removed from the crystal structure. Polar hydrogens were added, and Kollman charges were assigned to the receptor’s amino acids to compute partial charges. Compounds with strong affinity (≤ -7 kcal/mol) and multiple binding amino acid sites (≥ 5) were identified as candidate drugs which inhibited NlpD protein.

### Surface plasmon resonance (SPR) analysis

The CM5 sensor chip was activated immediately before coupling by mixing 400 mM EDC with 100 mM NHS. Activation was carried out at a flow rate of 10 μL/min for 420 s. β-Lactamase was diluted to 200 μg/mL in immobilization buffer and injected into the sample channel at a flow rate of 10 μL/min to achieve immobilization. Ketoconazole (KTZ) was prepared in the same analyte buffer at serial concentrations of 10, 5, 2.5, 1.25, 0.625, and 0.3125 μM. KTZ was injected into channels at 30 μL/min with an association phase of 60 s followed by a dissociation phase of 100 s. Both phases were conducted in the analyte buffer. Injections were repeated in ascending order of concentration for seven cycles. After each cycle, the sensor chip surface was regenerated with 10 mM Glycine-HCl at 30 μL/min for 30 s to remove the analyte before the next injection [77].

### Checkerboard assay

The synergistic activity between colistin and KTZ was evaluated using a broth microdilution checkerboard assay. An 8 × 8 drug-drug concentration matrix was prepared in 96-well plates by dispensing 100 μL Mueller-Hinton broth (MHB) per well. Colistin and KTZ were diluted along the abscissa and ordinate, respectively. Wells were inoculated with bacteria to a final density of 1 × 10^6^ CFU/mL and incubated at 37 °C for 18 h. The absorbance of each well at 600 nm was quantified by a microplate reader (Tecan Infinite E Plex). Fractional inhibitory concentration indices (FICI) were calculated as: FIC Index (FICI) = FICI_A_ + FICI_B_ = MIC_AB_/MIC_A_ + MIC_BA_/MIC_B_. FICI ≤ 0.5 demonstrates synergy.

### Safety evaluation

The hemolytic activity of KTZ was tested using sterile defibrinated sheep erythrocytes. Briefly, 8% erythrocytes were incubated with serial KTZ concentrations (0–256 μg/mL) at 37 °C for 1 h, with sterile PBS as blank control and ddH_2_O as positive control. After centrifugation at 3000 g for 10 min at 4 °C, 100 μL supernatant was transferred to a 96-well plate, and absorbance at 576 nm was measured using a Tecan Infinite M200 reader. Hemolysis rate was then estimated against controls.

The *in vivo* toxicity assessment model was conducted as follows. Five 7-week-old male BALB/c mice (22–25 g) were selected for the safety evaluation of KTZ. These mice were randomly divided into four groups, with two groups receiving an intraperitoneal injection of 30 mg/kg KTZ and another two groups receiving an intraperitoneal injection of PBS as a negative control. Body weight and survival rate were recorded during 1–14 d. At 2 and 14 d after treatment, 1 mL of blood was collected via cardiac puncture to detect serum biochemical indicators (ALT, AST, UREA, CREA) as well as blood ion levels (Na^+^, K^+^, Cl^−^). Meanwhile, the mice were sacrificed and their liver and kidneys were harvested to observe histopathological changes.

### Statistical analysis

Statistical tests were performed in GraphPad Prism version 8.0 software. Two group comparisons were analyzed using two-tailed Student’s t-test. Comparisons among three or more groups were analyzed by One-way ANOVA followed by an LSD post hoc test. Animal experiments were analyzed using the Mann-Whitney U-test. Unless otherwise noted, data are presented as mean ± SEM in plots with error bars. Statistical details, including *n* values, were provided in the figure legends. Significance levels are presented by numbers of asterisks: *P* < 0.05 (*), *P* < 0.01(**), *P* < 0.001(***), and ns, not significant.

## Supporting information

S1 Fig Analysis of transposon insertion sites in the genome of *S.* 4,[5],12:i:- in input and output libraries. (**A**) Transposon insertion site distribution of *S.* 4,[5],12:i:-. The indications of each circle from the outside are *S.* 4,[5],12:i:- CDS (+), *S.* 4,[5],12:i:- CDS (−), number of reads per insertion locus, and transposon insertion sites (green). (**B**) Insertion-site information for genes in the input and output libraries.
(TIF)

S2 Fig Construction of gene deficient strain. (**A**) Schematic diagram of constructing gene knockout strain via homologous recombination using the pRE112 suicide vector. (**B**) PCR verification of Δ*nlpD* mutants in *S.* 4,[5],12:i:-. (**C**) Sanger sequencing of *nlpD* gene deletion in *S.* 4,[5],12:i:-. (**D**) PCR verification and sanger sequencing of *torR, apt, and bcp* gene deletion in *S.* 4,[5],12:i:-. Quantitative analysis of phagocytosis rates (**E**) of RAW264.7 cells infected with WT strains and four mutant strains (Δ*nlpD*, Δ*torR*, Δ*apt*, and Δ*bcp*) and (**F**) intracellular survival capacity of WT strains and mutant strains (Δ*nlpD*, Δ*torR*, Δ*apt*, and Δ*bcp*) in RAW264.7 cells for 6 h. The number of bacteria released from the cells was quantified by plating the lysates. The quantity of mutant strains (Δ*nlpD*, Δ*torR*, Δ*apt*, and Δ*bcp*) were calculated relative to that of the WT (100%). Error bars represent SD; n = 3 independent experiments. Statistical analyses were performed by One-way ANOVA. *P < 0.05; **P < 0.01; ***P < 0.001; ns: not significant.
(TIF)

S3 Fig *nlpD* deletion in 14028S strain effectively attenuates bacterial replication both *in vitro* and *in vivo.* Quantitative analysis of phagocytosis rates (**A**) of RAW264.7 cells infected with 14028S, Δ*nlpD* and p*nlpD* strains and (**B**) intracellular survival ratio of 14028S, Δ*nlpD* and p*nlpD* strains under different concentrations of IFN-γ (0, 10, 20 ng/mL). Cells were infected with different strains at an MOI of 10 for 1 h and incubated for another 6 h. The number of bacteria released from the cells was quantified by plating the lysates. The quantity of Δ*nlpD* and p*nlpD* were calculated relative to that of the 14028S (100%). Error bars represent SD; n = 3 independent experiments. Statistical analyses were performed by Two-way ANOVA. ***P < 0.001; ns: not significant. (**C**) Comparison of organ colonization properties among the 14028S, Δ*nlpD* and p*nlpD* strains. The infection dose was 1 × 10^9^ CFU. Experiments were performed at 24 h post-infection. Log_10_ (CFU/g) represents the mean of six mice per group. Error bars represent SD. Data were obtained from two independent experiments. Statistical analyses were performed by Mann-Whitney U test. ***P < 0.001. (**D**) Pathological tissue section analysis of liver, spleen, and colon from mice infected with different strains (14028S, Δ*nlpD* and p*nlpD*) (10 x and 40 x magnification). Compared to the 14028S-infected and p*nlpD*-infected groups, Δ*nlpD*-infected mice exhibited reduced inflammatory cell infiltration and relatively preserved tissue architecture across all examined organs. (**E**) Cytokines expression (IL-1β, IL-6, TNF-α and IL-4) in liver, spleen, and cecum of mice was measured after 24 hpi following infection with 14028S, Δ*nlpD*, and p*nlpD* strains. Each dot represents an organ from an infected mouse. The baseline values were subtracted from all samples. Error bars represent SD; n = 3 independent experiments. Statistical analyses were performed by Two-way ANOVA. *P < 0.05; **P < 0.01; ***P < 0.001; ns: not significant.
(TIF)

S4 Fig *nlpD* deletion diminishes intracellular survival of *S.* Pullorum WF770. (**A**) PCR verification and sanger sequencing of *nlpD* gene deletion in *S.* Pullorum WF770. Quantitative analysis of phagocytosis rates (**B**) of HD11 cells infected with WT strains and Δ*nlpD* in *S.* Pullorum WF770 and (**C**) intracellular survival capacity of Δ*nlpD* strains of *S.* Pullorum WF770 in HD11 cells comparison to WT strains. Cells were infected with WT and Δ*nlpD* strains at an MOI of 10 for 1 h and incubated for another 6 h. The number of bacteria released from the cells was quantified by plating the lysates. The quantity of Δ*nlpD* was calculated relative to that of the WT (100%). Error bars represent SD; n = 3 independent experiments. Statistical analyses were performed by Student’s t-test. **P < 0.01.
(TIF)

S5 Fig Transcriptome analysis of 14028S and Δ*nlpD* strains in infected RAW264.7 cells for 6 h. (**A**) The principal component analysis (PCA) plot analysis of clustering patterns between 14028S and Δ*nlpD* strains infecting RAW264.7 cells at an MOI of 10 for 1 h and incubated for another 6 h using RNA-seq. Each colored dot represents an individual biological sample; n = 3 independent experiments. (**B**) The volcano plot of differentially expressed genes (DEGs) annotated for the WT versus Δ*nlpD* strains of 14028S. Log_2_ (FC) was calculated to account for the direction of expression difference. Genes with log_10_FDR values ≥ 1 and log_2_FC ≥ 1 or log_2_FC ≤ -1 were considered significantly different (red: up-regulated; blue: down-regulated). (**C**) The top 20 enriched GO terms were predominantly enriched in peroxiredoxin activity, response to hydroperoxide and peroxidase activity between WT versus Δ*nlpD* strains with a significant threshold of *P* < 0.05.
(TIF)

S6 Fig *nlpD* deletion in 14028S strain decreases the antioxidant capacity of bacteria. (**A**) Bactericidal curves of 14028S, Δ*nlpD* and p*nlpD* strains under different pH conditions (pH = 5, 7) at 6 and 12 h, while different concentrations of H_2_O_2_ (0, 2.5, 3 mM) at 12 and 24 h. Error bars represent SD; n = 3 independent experiments. (**B**) SOD enzyme activity of Δ*nlpD* strains was significantly reduced compared to WT strains at pH = 7 and pH = 5. The Total Superoxide Dismutase Assay Kit with WST-8 was used to measure SOD enzyme activity. Error bars represent SD; n = 3 independent experiments. Statistical analyses were performed by Two-way ANOVA. *P < 0.05; ***P < 0.001.
(TIF)

S7 Fig NlpD deficiency enhances LPS synthesis and TLR4 expression. (**A**) Relative mRNA Expression (Fold Change) of LPS synthesis-related genes of Δ*nlpD* vs WT strains quantified by RT-qPCR. Error bars represent SD; n = 3 independent experiments. Data were normalized to the reference gene *16S* and the control group was set as the baseline value of 1. (**B**) TLR4 and TLR2 proteins expression of RAW264.7 cells infected by WT or Δ*nlpD* strains (n = 4). (**C**) The quantitative analysis of gray value of (**B**) using FIJI software. Data were expressed as percentages normalized by CON group. Error bars represent SD; n = 4 independent experiments. Statistical analyses were performed by Two-way ANOVA. *P < 0.05; ***P < 0.001; ns: not significant. Quantitative analysis of phagocytosis rates (**D**) of BMDM cells infected with WT or Δ*nlpD* strains and (**E**) intracellular survival capacity of Δ*nlpD* mutant in BMDM cells in comparison with the WT strain for 6 h. The quantity of Δ*nlpD* was calculated relative to that of the WT (100%). Error bars represent SD; n = 3 independent experiments. Statistical analyses were performed by Student’s t-test. *P < 0.05; ***P < 0.001. (**F**) TLR4 and TLR2 proteins expression of BMDM cells infected by WT or Δ*nlpD* strains (n = 4). (**G**) The quantitative analysis of gray value of (**F**) using FIJI software. Data were expressed as percentages normalized by CON group. Error bars represent SD; n = 4 independent experiments. Statistical analyses were performed by Two-way ANOVA. ***P < 0.001; ns: not significant.
(TIF)

S8 Fig *nlpD* deletion promotes M1 polarization and enhances autophagy flux of BMDMs. (**A**) Expression levels of NF-κB pathway related proteins (NF-κB, p-NF-κB, IκB, and p-IκB) of uninfected BMDMs (CON) and cells infected by WT or Δ*nlpD* mutants for 6 h (n = 4). (**B**) The quantitative analysis of gray value of (**A**) using FIJI software. Data were expressed as percentages normalized by CON group. Error bars represent SD; n = 4 independent experiments. Statistical analyses were performed by One-way ANOVA. *P < 0.05; ***P < 0.001; ns: not significant. (**C**) Cytokines expression level (IL-1β, TNF-α, IL-4, and IL-10) of uninfected BMDMs (CON) and cells infected by WT or Δ*nlpD* mutants for 6 h. The baseline values were subtracted from all samples. Error bars represent SD; n = 3 independent experiments. Statistical analyses were performed by Two-way ANOVA. *P < 0.05; ***P < 0.001; ^##^*P* < 0.01; ^###^*P* < 0.001; ^&^*P* < 0.05; ^&&^*P* < 0.01; ^&&&^*P* < 0.001; ns: not significant. (**D**) Protein expression of iNOS (M1 marker), Arg-1 (M2 marker), and Hif-1α of uninfected BMDMs (CON) and cells infected by WT or Δ*nlpD* mutants for 6 h (n = 4). (**E**) The quantitative analysis of gray value of (**D**) using FIJI software. Data were expressed as percentages normalized by CON group. Error bars represent SD; n = 4 independent experiments. Statistical analyses were performed by One-way ANOVA. **P < 0.01; ***P < 0.001. (**F**) Immunoblot analysis of autophagy markers (LC3, p62, and Beclin 1) of uninfected BMDMs (CON) and cells infected by WT or Δ*nlpD* mutants for 6 h (n = 4). (**G**) The quantitative analysis of gray value of (**F**) using FIJI software. Data were expressed as percentages normalized by CON group. Error bars represent SD; n = 4 independent experiments. Statistical analyses were performed by One-way ANOVA. ***P < 0.001; ns: not significant.
(TIF)

S9 Fig Screening of siRNAs to silence Beclin 1 expression. The Beclin 1 protein expression level of RAW264.7 cells transfected with siRNA-1, siRNA-2 and siRNA-3 for 24 h (**A**) and 48 h (**B**), respectively. Data were expressed as percentages normalized by CON group. Error bars represent SD; n = 3 independent experiments. Statistical analyses were performed by One-way ANOVA. *P < 0.05; **P < 0.01; ***P < 0.001; ns: not significant. (**C**) The phagocytosis rates and intracellular survival rate of RAW264.7 cells infected by WT or Δ*nlpD* strains, transfected with or without siRNA-Beclin 1 for 48 h. The number of bacteria released from the cells was quantified by plating the lysates. The quantity of bacteria was calculated relative to that of the WT (100%). Error bars represent SD; n = 3 independent experiments. Statistical analyses were performed by One-way ANOVA. *P < 0.05; **P < 0.01.
(TIF)

S10 Fig NlpD deficiency elevates L-lactate contents. (**A**) The L-lactate content and (**B**) ATP concentrations in RAW264.7 cells infected by either WT or Δ*nlpD* strains, pretreated with or without 2-DG (5 mM) for 1 h. Error bars represent SD; n = 3 independent experiments. Statistical analyses were performed by Two-way ANOVA. *P < 0.05; **P < 0.01; ***P < 0.001. (**C**) The intracellular survival capacity of RAW264.7 cells infected by WT strains or Δ*nlpD* strains for 6 h, pretreated with or without 2-DG (5 mM) for 1 h. The quantity of Δ*nlpD* was calculated relative to that of the WT (100%). Error bars represent SD; n = 3 independent experiments. Statistical analyses were performed by Two-way ANOVA. **P < 0.01; ns: not significant.
(TIF)

S11 Fig High-throughput screening of FDA-approved drugs targeting NlpD. (**A**) The intracellular survival capacity of WT and Δ*nlpD* strains in RAW264.7 cells treated by different candidate NlpD-targeting drugs (Perphenazine, Ketoconazole, Isradipine, Obeticholic Acid, Reserpine, Pazopanib, Sulfasalazine, Lovastatin) identified by molecular docking. Error bars represent SD; n = 3 independent experiments. Statistical analyses were performed by Two-way ANOVA. *P < 0.05; **P < 0.01; ***P < 0.001; ns: not significant. (**B**) Relative mRNA expression (Fold Change) of *nlpD* in WT and Δ*nlpD* strains treated with or without FDA drugs (Ketoconazole, Obeticholic Acid, Pazopanib) for 6 h measured by RT-qPCR. Data were normalized to the reference gene *16S* and the control group was set as the baseline value of 1. Error bars represent SD; n = 3 independent experiments. Statistical analyses were performed by One-way ANOVA. **P < 0.01; ***P < 0.001. (**C**) RAW264.7 cells viability treated by 3 FDA drugs (Ketoconazole, Obeticholic Acid, Pazopanib) varying different concentrations (0, 3, 6, 12, 25, 50, 100 μM) at 12 h. Data were expressed as percentages normalized by 0 μM group. Error bars represent SD; n = 3 independent experiments. (**D**) Intracellular survival rates of WT and Δ*nlpD* strains in RAW264.7 cells treated by Ketoconazole (50 μM), Obeticholic Acid (25 μM) and Pazopanib (25 μM). Error bars represent SD; n = 3 independent experiments. Statistical analyses were performed by Two-way ANOVA. *P < 0.05; **P < 0.01; ***P < 0.001. (**E**) The intracellular survival capacity of HD11 cells infected with WT strains and Δ*nlpD* strains of *S.* Pullorum WF770 for 6 h with or without KTZ (10 μM) treatment. The quantity of Δ*nlpD* was calculated relative to that of the WT (100%). Error bars represent SD; n = 3 independent experiments. Statistical analyses were performed by Two-way ANOVA. ***P < 0.001.
(TIF)

S12 Fig Synergistic activity between KTZ and colistin against *S*. 4,[5],12:i:-. (**A**) SDS-PAGE of purified NLPD protein. (**B**) Checkerboard assays revealed synergistic activity between KTZ and colistin against *S.* 4,[5],12:i:- *in vitro*, with a fractional inhibitory concentration index (FICI) of 0.5. (**C**) Time-killi curves of KTZ in combination with colistin against *S.* 4,[5],12:i:-. Bacteria were treated with colistin (0.5 μg/mL), KTZ (125 μg/mL), or their combination (0.5 μg/mL colistin +125 μg/mL KTZ). Viable bacterial counts were determined at 0, 6, and 12 h by colony counting and expressed as log_10_ CFU/mL. Error bars represent SD; n = 3 independent experiments.
(TIF)

S1 Table Genes with ≤ 1 insertion in output library from TraDIS screening. (XLSX)

S2 Table Sequences of Beclin 1-siRNA. (XLSX)

S3 Table FDA-approved drug candidates targeting NlpD identified by molecular docking. (XLSX)

S4 Table PCR primer sequences for constructing deletion mutants. (XLSX)

S5 Table RT-qPCR primer sequences used in this study. (XLSX)

S6 Table Antibodies used in immunoblotting analysis in this study. (XLSX)
